# eDNA, Amyloid Fibers and Membrane Vesicles Identified in *Pseudomonas fluorescens* SBW25 Biofilms

**DOI:** 10.3390/ijms232315096

**Published:** 2022-12-01

**Authors:** Olena V. Moshynets, Ianina Pokholenko, Olga Iungin, Geert Potters, Andrew J. Spiers

**Affiliations:** 1 Institute of Molecular Biology and Genetics, National Academy of Sciences of Ukraine, 03143 Kyiv, Ukraine; 2 Department of Biotechnology, Leather and Fur, Kyiv National University of Technologies and Design, 01011 Kyiv, Ukraine; 3 Antwerp Maritime Academy, 2030 Antwerp, Belgium; 4 Department of Bioscience Engineering, University of Antwerp, 2000 Antwerp, Belgium; 5 School of Applied Sciences, Abertay University, Dundee DD1 1HG, UK

**Keywords:** air–liquid interface, amyloid fibers, biofilm, eDNA, EPS, outer-membrane vesicles, *Pseudomonas*

## Abstract

*Pseudomonas fluorescens* SBW25 is a model soil- and plant-associated bacterium capable of forming a variety of air–liquid interface biofilms in experimental microcosms and on plant surfaces. Previous investigations have shown that cellulose is the primary structural matrix component in the robust and well-attached Wrinkly Spreader biofilm, as well as in the fragile Viscous Mass biofilm. Here, we demonstrate that both biofilms include extracellular DNA (eDNA) which can be visualized using confocal laser scanning microscopy (CLSM), quantified by absorbance measurements, and degraded by DNase I treatment. This eDNA plays an important role in cell attachment and biofilm development. However, exogenous high-molecular-weight DNA appears to decrease the strength and attachment levels of mature Wrinkly Spreader biofilms, whereas low-molecular-weight DNA appears to have little effect. Further investigation with CLSM using an amyloid-specific fluorophore suggests that the Wrinkly Spreader biofilm might also include Fap fibers, which might be involved in attachment and contribute to biofilm strength. The robust nature of the Wrinkly Spreader biofilm also allowed us, using MALDI-TOF mass spectrometry, to identify matrix-associated proteins unable to diffuse out of the structure, as well as membrane vesicles which had a different protein profile compared to the matrix-associated proteins. CLSM and DNase I treatment suggest that some vesicles were also associated with eDNA. These findings add to our understanding of the matrix components in this model pseudomonad, and, as found in other biofilms, biofilm-specific products and material from lysed cells contribute to these structures through a range of complex interactions.

## 1. Introduction

Bacterial biofilms are complex structures using a variety of polymers as structural elements (matrix), including polysaccharides (EPS), proteins, and extracellular DNA [1]. eDNA is involved in cell adhesion during the early stages of biofilm development and plays a key structural role in mature biofilms [2,3,4,5,6]. eDNA was first visualized in solid surface–liquid (S–L) interface (i.e., submerged) biofilms produced by the opportunistic human pathogen, *Pseudomonas aeruginosa* PA01 [7], where it aids the initial adhesion of cells to surfaces, promotes cell aggregation, and interacts with EPS to form fiber-like networks stabilizing the biofilms forming at different interfaces [8,9,10]. eDNA is now known to be produced by a range of benign and pathogenic species, including *Bacillus subtilis* [11], *Escherichia coli* [12], *Salmonella enterica* [13], *Staphylococcus aureus* and *S. epidermis* [14,15], *Streptococcus mutans* and *S. pneumoniae* [16,17], etc., and is widespread in aquatic environments, sediments, and soil [4]. However, the origin of eDNA is varied [3,4] and here we do not infer whether eDNA secretion or release is deliberate at the single-cell or population level (i.e., is produced), or whether it is an indirect and advantageous consequence of cell death and lysis.

We were involved in the early description of the air–liquid (A–L) interface biofilm produced by the Wrinkly Spreader, a novel biofilm-forming mutant of the model soil- and plant-associated pseudomonad, *P. fluorescens* SBW25, isolated from experimental evolution microcosms [18,19]. A–L interface biofilms are sometimes referred to as pellicles [10] and the Wrinkly Spreader biofilm is not technically floating but is retained at the surface of the liquid column by attachment to the vial walls at the meniscus and hydrophobicity [20,21]. The Wrinkly Spreader biofilm is a very robust viscoelastic structure compared to the weaker Viscous Mass biofilm produced by wild-type *P. fluorescens* SBW25 when induced with exogenous Fe^3+^ [22,23], though both utilize partially acetylated cellulose as the primary structural matrix component [20,21,22,23,24]. In the Wrinkly Spreader biofilm, cellulose fibers interact with liposaccharide (LPS) and an unidentified attachment factor, possibly poly-N-acetylglucosamine (PNAG) and/or Fap amyloid fibers [19,25,26], to contribute to the biofilm structure and distinctive colony morphology [21]. *P. fluorescens* SBW25 also produces alginate [27,28] and possibly has the potential to produce other EPS, but these have not yet been implicated in biofilm formation. In other biofilms, amyloid fibers, PNAG, and other EPS are known to interact with eDNA [1,2,3,4,5,8], and interstitial fluids also include a variety of proteins, flagella, fimbriae, and pili, as well as membrane vesicles (MVs) produced by metabolically active, stressed, or lysed cells [1,5,6,8,9,29,30,31,32].

Although our description of the Wrinkly Spreader biofilm was contemporaneous with the first report of biofilm eDNA [7], we have never investigated *P. fluorescens* SBW25 biofilms for the presence of eDNA. Several other pseudomonad species are known to produce eDNA, such as *P. chlororaphis* 30–84 [33] and *P. putida* KT2440 [34] in A–L interface biofilms. *P. putida* KT2440 also produces cellulose matrix-based biofilms and Wrinkly Spreader-like mutants [35,36,37,38,39], as does *Pseudomonas* sp. FW1 [40] in a S-L interface biofilm, and *P. fluorescens* B/S_1000_ in a slime [41]. Fourier-transform infrared spectroscopy spectra suggest eDNA, besides nanocellulose, may be in other pseudomonad A–L interface biofilms, e.g., [42,43,44]. However, we are not aware of any other reports of eDNA for other *P. fluorescens* model strains, including CHA0, Pf0-1 and Pf5, or for the plant pathogen, *P. syringae* DC3000, which is capable of producing cellulose matrix-based A–L interface biofilms [35,43]. Both the *Pseudomonas* genus and *P. fluorescens* species are highly diverse [44,45,46,47,48], and the expression of biofilm EPS by pseudomonads is also highly varied [6,8,49], so it is possible that eDNA is not a common feature of these biofilms.

The aim of this work was to better understand the matrix composition of the Wrinkly Spreader biofilm and to determine whether it also included amyloid fibers and eDNA which have reported for other pseudomonad biofilms [7,25,33,34,40,41,50]. Although the Viscous Mass biofilm produced by wild-type *P. fluorescens* SBW25 is considerably more difficult to work with because it is such a fragile structure [22,23], we have included it in our investigation to see how varied the matrix composition might be within this pseudomonad strain. Our results confirm the presence of amyloid fibers and eDNA, and DNase I treatments demonstrate that eDNA is likely to be important during the early stages of biofilm development. Using MALDI-TOF, we have also visualized Wrinkly Spreader membrane vesicles and identified proteins from the matrix, as well as biofilm and planktonic vesicles. These findings have advanced our understanding of the matrix composition of this important model biofilm system and demonstrate that *P. fluorescens* SBW25 biofilms are structurally more complex than originally reported [20,21,22,23,24].

## 2. Results

### 2.1. Visualization of eDNA in Viscous Mass and Wrinkly Spreader Biofilms

**Figure 1:**
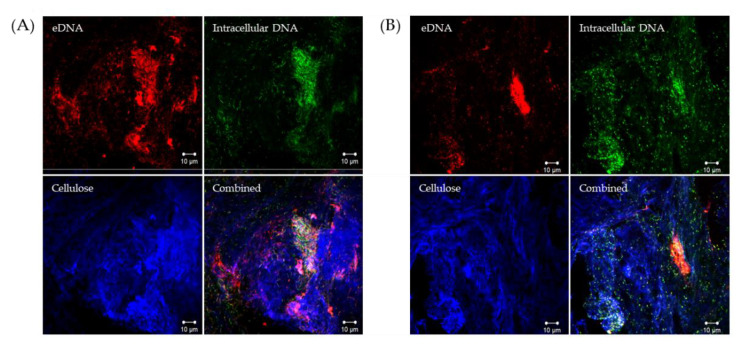
*P. fluorescens* SBW25 biofilms include eDNA. Confocal laser scanning microscopy (CLSM) was used to visualize eDNA, intracellular DNA, and cellulose in 24 h biofilms. Shown here are single-channel and combined CLSM images from Viscous Mass (**A**) and Wrinkly Spreader (**B**) biofilms. Propidium iodide (red channel) was used to visualize eDNA, CYTO9 (green channel) to visualize the intracellular DNA of live cells, and Calcofluor white (blue channel) to visualize cellulose, respectively, and all three channels are combined in the bottom-right image. The scale bars indicate 10 µm.

Confocal laser scanning microscopy (CLSM) was used to determine whether Viscous Mass biofilms produced by wild-type *P. fluorescens* SBW25 and Wrinkly Spreader mutant biofilms included eDNA as part of the matrix. We used the membrane-impermeable DNA-binding stain, propidium iodide, to visualize eDNA, plus the membrane-permeable counterstain, SYTO9, to visualize intracellular DNA from metabolically active (live) cells (the propidium iodide/SYTO9 combination is the basis of the ‘live/dead’ staining method [51], and Calcofluor white to visualize the cellulose matrix previously identified in Viscous Mass and Wrinkly Spreader biofilms [20,22,24,52]. CLSM images clearly showed the presence of eDNA in both biofilms, where it appeared to co-localize with cellulose above dense regions of active cells (Figure 1). Like cellulose, eDNA was present in thinner films and large, denser structures, but it is not clear whether the two are intermixed or occupy separate volumes above the surface of the cells, or whether eDNA and cellulose retain physical links with the secreting and/or lysing cells.

**Figure 2:**
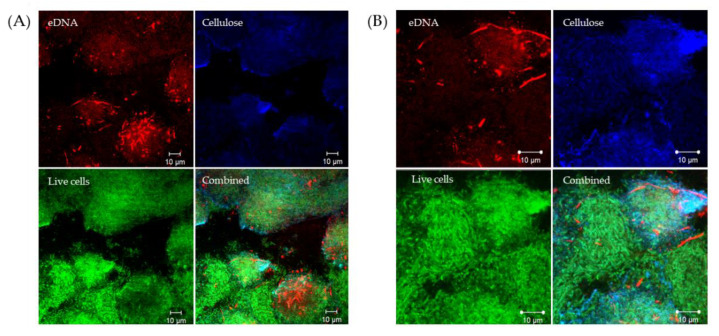
eDNA and cellulose accumulate above masses of cells in *P. fluorescens* SBW25 biofilms. Confocal laser scanning microscopy (CLSM) was used to visualize eDNA and cellulose against a backdrop of GFP-expressing metabolically active Wrinkly Spreader (WS-GFP) mutant cells in three-day-old biofilms. Shown here are single-channel and combined CLSM images of two sections of a Wrinkly Spreader biofilm (**A**,**B**). Propidium iodide (red channel) was used to visualize eDNA, GFP (green channel) to visualize live cells, and Calcofluor white (blue channel) to visualize cellulose, respectively, and all three channels are combined in the bottom-right image. The scale bars indicate 10 µm.

We obtained further evidence of eDNA in Wrinkly Spreader biofilms using a Green fluorescent protein (GFP) labeled Wrinkly Spreader mutant (WS-GFP [52]), instead of SYTO9, to mark live cells and avoid any possible contribution by intracellular DNA in dead, but intact, cells to the eDNA signal (GFP and SYTO9 signals have been shown to co-localize, e.g., [53]). CLSM images of these GFP–WS biofilms confirmed the presence of eDNA and supported our impression that both cellulose and eDNA were accumulating above the active cells (Figure 2). Further work would be needed to determine differences in eDNA spatial distribution and heterogeneity between the two biofilm types.

**Figure 3:**
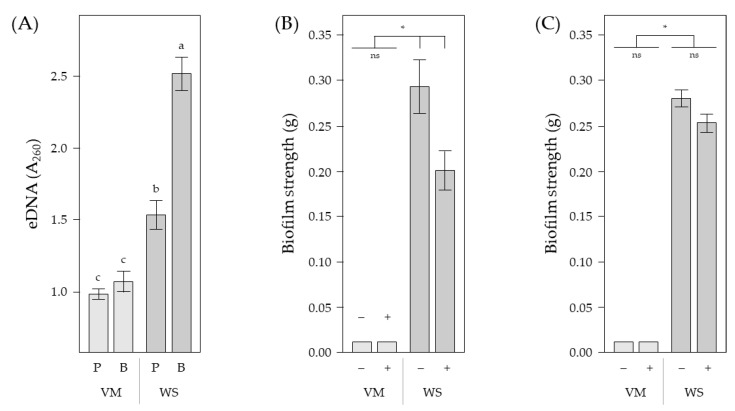
Quantitative measurements of eDNA and the impact it has on biofilm strength. Spectrometry was used to measure eDNA recovered from planktonic cultures and biofilms, and small glass balls to determine biofilm strength. Shown here are eDNA (A_260_) levels (**A**) in 24 h planktonic and three-day-old biofilm samples (B, biofilm sample; P, planktonic (shaken) culture sample; VM, Viscous Mass; WS, Wrinkly Spreader). Biofilm strengths (grams) are shown (**B**) for three-day-old biofilms which developed in microcosms with DNase I before assay, and (**C**) for three-day-old biofilms treated with DNase I for 3 h before assay (+, with; −, without). Means (*n* = 6–8) ± SE are shown. Differences between means (**A**) were determined for each set of measurements by one-way ANOVA and post hoc Tukey–Kramer HSD tests. Means sharing the same letter are not significantly different (α = 0.05). Differences between means (**B**,**C**) were determined by the Wilcoxon method. Means linked by an asterisk are significantly different (*p* < 0.05) (ns, not significantly different).

Although Propidium iodide/SYTO9 counterstaining or similar combinations, such as Propidium iodide/GFP or Propidium iodide/SYBR Green, is commonly used to visualize eDNA in biofilms (e.g., [34,51,54]), it is not always clear whether the eDNA signal is coming from extracellular DNA or from DNA within damaged or lysed cells, especially when cells may be stressed. We therefore sought confirmation of eDNA using two complementary approaches. First, we isolated eDNA from planktonic cultures and biofilms by removing cells and cellulose by centrifugation, with eDNA levels measured by spectrometry (A_260_) [55]. We were able to detect significantly higher levels of eDNA from Wrinkly Spreader biofilms compared to planktonic cultures (1.6×; Tukey–Kramer HSD, α = 0.05) (Figure 3A). Although the eDNA levels in Wrinkly Spreader biofilms were significantly higher than in Viscous Mass biofilms (2.3×), no significant difference in eDNA levels were observed between Viscous Mass biofilms and planktonic cultures (Tukey–Kramer HSD, α = 0.05), perhaps because it was harder to separate free eDNA from the more fragile biofilm [22,23] and/or less eDNA is produced in this biofilm.

### 2.2. Role of eDNA in Biofilm Strength and Attachment Levels

We then assessed the impact DNase I treatment (after [34,40,56] etc.) might have on strength with the expectation that it might contribute to the strength of mature biofilms, and/or was important for biofilm development, perhaps at the early stage of cell attachment to the microcosm vial walls. As the Viscous Mass and Wrinkly Spreader biofilms cover the air–liquid (A–L) interface, strength measurements (grams), which form part of the combined biofilm assay [57], are readily undertaken by placing a series of small glass balls on the upper surface until the biofilm rips apart or sinks. Using this approach, we found a significant reduction in the strength of Wrinkly Spreader biofilms when they were allowed to develop over three days in the presence of DNase I (1.5×; Wilcoxon, *p* = 0.02) (Figure 3B) (younger biofilms are simply too weak to be able to measure). Again, no significant difference was seen in Viscous Mass biofilm strength (Wilcoxon, *p* = 1.00) (Figure 3C), though it is difficult to measure the strength of these comparatively fragile biofilms [22,23]. It was also notable that the treatment of mature Wrinkly Spreader biofilms with DNase I for three hours did not significantly reduce strength (Wilcoxon, *p* = 0.12), suggesting that eDNA may only play an important role during the early phases of biofilm development, and/or it is more protected by the binding of proteins or interactions with other EPS in mature biofilms.

We added exogenous low-molecular-weight (LMW) and high-molecular-weight (HMW) DNA to microcosms (after [58]) to investigate the impact this might have on the development of Viscous Mass and Wrinkly Spreader biofilms over three days. In addition to the biofilm strength assay we previously used, we also determined biofilm attachment using crystal violet dye to stain bacterial cells adhering to the microcosm vial walls at the meniscus ([20,57], adapted from the procedure established by [59]). After washing to remove excess dye, crystal violet was then eluted with ethanol and absorbance (A_570_) measured to determine attachment levels. Finally, we measured final biomass levels using optical density (OD_600_) measurements to check whether our experimental treatments affected growth, as biofilm strength and attachment levels might be correlated with growth (i.e., faster growing cultures produce stronger and better attached biofilms).

We use a general linear mixed modelling approach to model biofilm strength, attachment levels, and final microcosm biomass (i.e., growth) as response variables with *biofilm type* (Viscous Mass and Wrinkly Spreader), *DNA treatment* (Controls with no exogenous DNA, LMW, and HMW), *DNA concentration* (0, 100, 300, and 600 µg/mL), and *replicate* as effects, and *biofilm type* × *DNA treatment* as an interaction (see Table S1 for model details and effects tests). This identified *biofilm type* as a significant effect (*p* < 0.01), as expected, because the Wrinkly Spreader biofilm is a stronger and better-attached structure, which supports a greater number of cells at the A–L interface and a higher microcosm biomass than the Viscous Mass biofilm. *DNA treatment* was also a significant effect (*p* < 0.01), but not *DNA concentration* (*p* = 0.21–0.92), suggesting that exogenous DNA added to the microcosms had an effect across treatments. Microcosms containing HMW DNA had final biomass levels 1.1× higher, but 0.6× lower biofilm strengths and 0.5× lower attachment levels than the control microcosms or those with LMW DNA (LSMeans Differences Tukey HSD, α = 0.05). The lack of a significant *DNA concentration* effect suggests that 100 µg/mL exogenous DNA saturated all binding sites and/or formed a sufficient gel-like network, and that additional DNA made no significant difference. Finally, we note that the *biofilm type* × *DNA treatment* interaction was significant in all models (*p* < 0.01).

**Figure 4:**
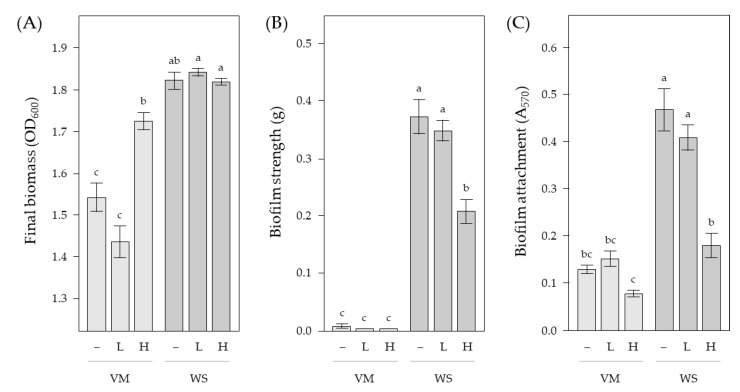
Exogenous DNA affects *P. fluorescens* SBW25 biofilm development. Low-molecular-weight (LMW) and high-molecular-weight (HMW) DNA was added to microcosms and Viscous Mass (VM) and Wrinkly Spreader (WS) biofilms allowed to develop for three days before assay. Shown here are the final microcosm biomass (OD_600_) (**A**), biofilm strength (grams) (**B**), and attachment (A_570_) (**C**) measurements (–, no exogenous DNA added; L, LMW DNA added; H, HMW DNA added). Means (*n* = 8–24) ± SE are shown. Differences between means were determined for each set of measurements by one-way ANOVA and post hoc Tukey–Kramer HSD tests. Means sharing the same letter are not significantly different (α = 0.05).

As no significant DNA concentration effect was seen in our models, we pooled the exogenous DNA treatments together (i.e., controls with no exogenous DNA, HMW DNA and LMW DNA) and used an analysis of variance (ANOVA) approach with post hoc Tukey–Kramer HSD tests to determine differences in final microcosm biomass, biofilm strength, and attachment levels (Figure 4). This confirmed our modelling results and shows more clearly the differences in response to exogenous DNA by the Wrinkly Spreader and Viscous Mass biofilms. We interpret these findings to mean that HMW DNA negatively affects the development of Wrinkly Spreader biofilms by reducing strength and attachment levels, without affecting final biomass levels. In contrast, HMW DNA increases the final biomass of Viscous Mass microcosms, perhaps by supporting greater levels of biofilm development, though this is not supported by significantly increased measurements of biofilm strength or attachment levels. However, increases in attachment and strength may be very hard to quantify given the very fragile nature of the Viscous Mass biofilm.

During the biofilm attachment assays, we noticed that Wrinkly Spreader microcosms containing LMW DNA retained considerably more crystal violet after the elution stage compared to the control microcosms or those with HMW DNA added (Figure S1A) (this retention of dye is not the result of limited washing, e.g., [60]; after staining, we wash the microcosm vials three times before elution with ethanol, which normally removes all of the dye from the meniscus region). The retention of the crystal violet stain may reflect a ring of densely attached cells at the meniscus which absorbs more dye and releases it more slowly than is normally the case. We investigated this with a second elution stage using acetic acid (a commonly used alternative to elution with ethanol [61]) followed by absorbance measurements. This allowed us to determine that Wrinkly Spreader biofilms developing in microcosms with LMW DNA release more crystal violet in the acetic acid elution than either the control microcosms or those with HMW DNA (2.0 and 2.4×, respectively; Wilcoxon, *p* < 0.01) (Figure S1B). Similarly, significantly more stain was also eluted from Viscous Mass microcosms incubated with LMW DNA with acetic acid compared to microcosms with HMW DNA (1.2×; Wilcoxon, *p* < 0.01), though there was no significant difference compared to the control microcosms (Wilcoxon, *p* = 0.18).

The additional elution of crystal violet with acetic acid does not change our understanding of the impact of HMW DNA on the attachment levels of Viscous Mass and Wrinkly Spreader biofilms, but it does reveal a more significant role for LMW DNA which was missed in our earlier attachment assays. LMW DNA appears to enhance bacterial cell attachment to the microcosm vial walls at the meniscus, allowing greater levels of attachment, and/or the development of a denser layer of attached cells from which the A–L interface biofilms then develop out over the liquid surface. However, we do not know why LMW and HMW DNA have such different effects. Interestingly, we have observed large pockets of eDNA, visualized by CLSM in preliminary experiments with Experimental Stain 986, a tetrahydronaphthalenylidene-benzothiazolium derivative, in Wrinkly Spreader biofilms incubated with LMW DNA (Figure S2), but not with HMW DNA, which suggests that the physical properties of the exogenous DNA underlie the differences seen in bacterial attachment.

### 2.3. Viscous Mass and Wrinkly Spreader Biofilms include Amyloid Fibers

**Figure 5:**
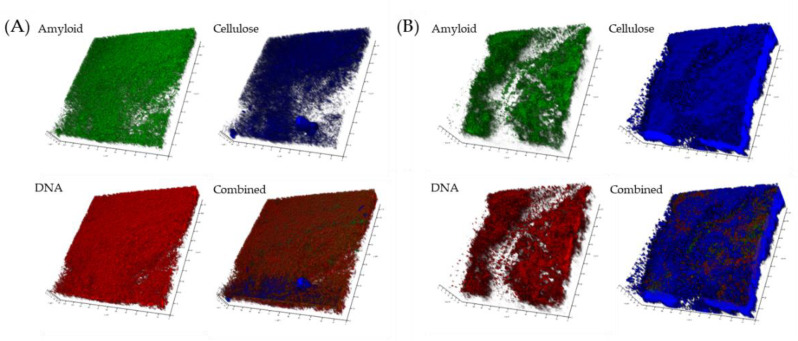
*P. fluorescens* biofilms include amyloid fibers. Confocal laser scanning microscopy (CLSM) was used to visualize amyloid fibers in three-day-old Viscous Mass and Wrinkly Spreader and biofilms. Shown here are single-channel and combined CLSM images of Viscous Mass (**A**) and Wrinkly Spreader (**B**) three-day-old biofilms. AmyGreen (green channel) was used to visualize amyloid fibers, Calcofluor white (blue channel) to visualize cellulose, and ethidium bromide (red channel) to visualize total DNA as a proxy for live cells, respectively, and all three channels are combined in the bottom-right images. Note the low signal region running down the Wrinkly Spreader image. The dimensions of the images are 115 × 115 × 20/35 µm.

We used CLSM and the fluorescent β-ketoenole AmyGreen dye [62,63] to investigate whether Fap fibrils were produced in Viscous Mass and Wrinkly Spreader biofilms. In both biofilms (Figure 5), a strong AmyGreen signal was observed and appears to be co-localized with the ethidium bromide signal used to visualize total DNA as a proxy for cell numbers, rather than with cellulose which stretches across rips seen in the Wrinkly Spreader sample. An analysis of the total pixel sums for each of the AmyGreen, Calcofluor white, and ethidium bromide channels (*n* = 3 images) suggests no significant difference in amyloid fibers or cell numbers between biofilms (*t*-tests, *p* = 0.31 and 0.42, respectively) though cellulose levels in the Wrinkly Spreader biofilm were significantly higher than in the Viscous Mass biofilm (4×, *t*-test, *p* = 0.04). However, the relative production of amyloid fibers requires further corroboration with the use of *fap* deletion mutants and the testing of samples from the early attachment stage to mature biofilms.

### 2.4. Biofilm Membrane Vesicles and Matrix-Associated Proteins

The biofilm interstitial fluid is also known to contain membrane vesicles (MVs) and proteins secreted from metabolically active cells or released by stressed and/or dying cells [29,30,32]. Biofilm vesicles and matrix-associated proteins are generally investigated by removing cells and large debris by centrifugation, with vesicles isolated by filtration, and matrix-associated proteins by pelleting the matrix with further centrifugation, resuspension, and solubilization in an appropriate buffer (after [17] etc.). Although we were interested in comparing vesicles and matrix-associated proteins from both Wrinkly Spreader and Viscous Mass biofilms, this was only practical for the Wrinkly Spreaders which produced sufficiently robust biofilms to allow handling and a clear separation of cells from the biofilm matrix and vesicles (similarly, cells could be separated from vesicles from planktonic samples).

**Figure 6:**
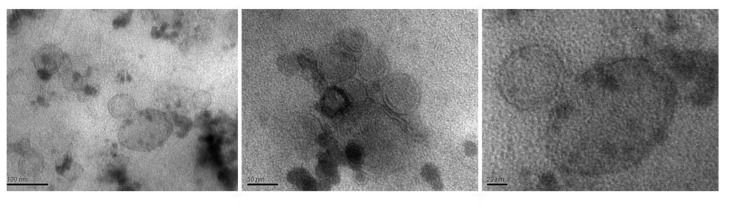
*P. fluorescens* SBW25 Wrinkly Spreader biofilms contain membrane vesicles. Shown here are three transmission electron microscopy (TEM) images of increasing magnification of membrane vesicles (MVs) isolated from three-day-old Wrinkly Spreader biofilms. The scale bars indicate 100 nm, 50 nm, and 20 nm (**left** to **right**).

We were able to isolate vesicles from three-day-old Wrinkly Spreader biofilms and 24 h planktonic cultures using centrifugation, followed by passage through a 0.2 µm pore-size filter to select for vesicles [17]. Although considerable variation in shape and size was observed, transmission electron microscopy (TEM) images suggested a diameter of ~50 nm for the more circular structures, and in the higher magnification images, double membranes were clearly seen (Figure 6). Further analysis of biofilm vesicle samples by CLSM indicates a co-localization of DNA-staining ethidium bromide and membrane-specific Vybrant DiO [64] signals, and DNase I treatment of these preparations removes all DNA, suggesting that it is associated with the outer surface of the MVs and is not internalized (Figure S3).

We also investigated Wrinkly Spreader biofilm matrix-associated proteins by repeatedly washing three-day-old biofilms over seven days to remove soluble and poorly attached proteins, and pre-incubating samples with SDS and urea to release strongly attached proteins before SDS-PAGE (Figure S4). This demonstrated that significant amounts of protein are retained by the matrix and are prevented from freely diffusing out of the structure (we were unable to extract Viscous Mass biofilm proteins in a similar manner, as any physical disturbance reduces the biofilm to a viscous liquid that cannot then be manipulated).

**Figure 7:**
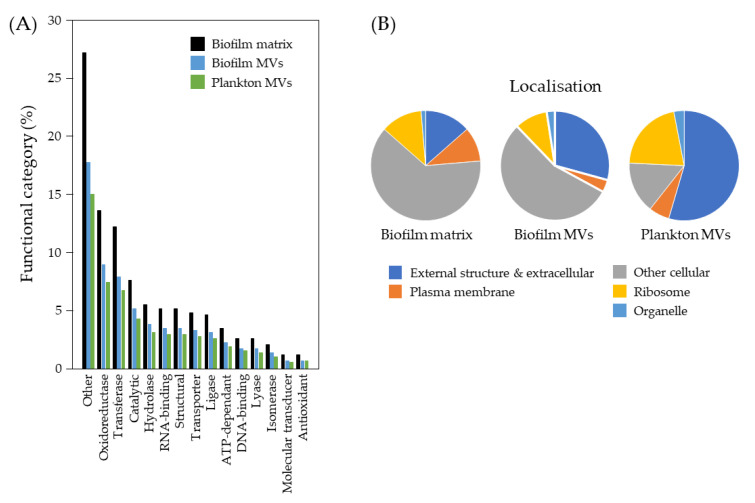
*P. fluorescens* SBW25 Wrinkly Spreader biofilm matrix and membrane vesicles have different protein profiles. Proteins associated with the biofilm matrix and membrane vesicles (MVs) isolated from biofilms and plankton (shaken cultures) were identified by MALDI-TOF mass spectrometry and categorized according to GO functional activities and predicted localization. Shown here (left) are the top 15 functional (**A**) and five cellular localization (**B**) categories for proteins identified from biofilm matrix samples, biofilm vesicle and planktonic vesicle samples.

We then used MALDI-TOF mass spectrometry to identify the most abundant proteins with emPA1 ≥ 1 in the Wrinkly Spreader biofilm matrix, and biofilm and planktonic membrane vesicle samples. This identified 94 proteins from the biofilm matrix (52 of 319 proteins with emPA1s of 0.03–10.9), biofilm vesicles (31 of 222 proteins with emPA1s of 0.03–5.36) and planktonic vesicles (13 of 166 proteins with emPA1s of 0.07–2.28) (see Table S2 for all identified proteins). Although some of these are homologs of proteins found in *P. aeruginosa* PA01 biofilms and vesicles (e.g., [29,65,66,67]), we are primarily interested in determining whether the protein profiles differ significantly between these samples. For each protein we extracted the GO functional annotation and predicted localization from UniProt, with details summarized in Figure 7. The source of the proteins and top 15 functional annotations are not independent (3 × 15 contingency table, χ^2^, *p* < 0.02), and pairwise tests confirm that, in each case, protein profiles differed between biofilm matrix, biofilm vesicle, and plankton vesicle samples (2 × 15 contingency tables, χ^2^, *p* ≤ 0.01). Similarly, protein source and the top five predicted localizations are not independent (3 × 5 and 2 × 5 contingency tables, χ^2^, *p* ≤ 0.03). Proportionally, the biofilm matrix appears to contain more cellular and ribosomal proteins than either of the two vesicle samples, suggesting that most matrix proteins are released by cell lysis rather than by a more controlled process, as is the case of membrane vesicle biogenesis. The biofilm vesicles also appear to contain proportionally more cellular proteins and fewer proteins associated with external structures or secreted proteins than the planktonic vesicles. However, further work would be needed to be able to establish which proteins might be unique to certain types of membrane vesicle or only associated with the biofilm matrix.

## 3. Discussion

The Wrinkly Spreader biofilm was the first to be reported for a pseudomonad utilizing cellulose as the main matrix component [20,24]. Although cellulose production is now known to be common amongst the pseudomonads, especially in the fluorescent species which also readily form air–liquid (A–L) interface biofilms [35], biofilm literature is dominated by a relatively few *P. aeruginosa* strains, producing liquid–solid surface (L–S) interface biofilms utilizing multiple EPS. Here, we demonstrate that eDNA and amyloid fibers are present in the matrix of Viscous Mass and Wrinkly Spreader biofilms, and that eDNA has a structural role, at least in the physically more robust Wrinkly Spreader biofilm.

We have used confocal laser scanning microscopy (CLSM) with propidium iodide to visualize eDNA in Viscous Mass and Wrinkly Spreader biofilms following standard protocols, and confirmed the presence of eDNA using a GFP-labelled Wrinkly Spreader mutant to visualize metabolically active cells, rather than CYTO9 to stain intracellular DNA. DNase I treatments indicate eDNA plays a structural role in the Wrinkly Spreader biofilm, though the fragile nature of the Viscous Mass biofilm did not allow us to confirm a similar structural role in this biofilm.

Earlier research has shown that the Wrinkly Spreader biofilm and phenotype is dependent on the production of partly acetylated cellulose and an unidentified attachment factor, possibly PNAG and/or Fap amyloid fibers, and on interactions with liposaccharide (LPS), cells, and debris [19,20,21,24,25,26]. Since a cellulose-deficient Wrinkly Spreader mutant does not produce a biofilm in experimental microcosms [24], we can conclude that eDNA is not the only critical component of biofilm formation. Similarly, cellulose matrix–based A–L interface biofilms produced by *P. putida* that also contain eDNA still develop even after DNase I treatment [34]. Nonetheless, we have shown that the addition of exogenous DNA affects Wrinkly Spreader biofilm development, with LMW and HMW DNA having different impacts on biofilm attachment levels. eDNA absorbs to the surface of cells in long loop-like structures [2] and forms a conditioning layer on glass [68]. Although we do not know the basis for the different impact of exogenous LMW and HMW DNA, DNA entanglement might favor LMW DNA to produce relatively small but dense network structures which aid cell attachment and the early development of the biofilm. In contrast, HMW DNA might produce looser but larger structures which are more sensitive to physical disturbance and distort or break away from the main body of the biofilm. The presence of non-canonical Hoogsteen base-pairing and G-quadruplex (square planar) structures in the eDNA of *P. aeruginosa* PA01 RSCV mutant biofilms [69] illustrates just how complex eDNA networks become in maturing biofilms (Holliday junction recombination intermediates have also been observed in other eDNA [70]). DNase I treatment of developing Wrinkly Spreader biofilms substantially reduces biofilm strength suggesting that eDNA is important early in biofilm formation but has little impact on mature biofilms where it might also be protected from degradation by non-canonical base pairing and association with EPS fibers, proteins, and other matrix components.

The importance of eDNA during the early stages of biofilm development is supported by our exogenous DNA experiments which suggest that LMW DNA increases cell–wall or cell–cell attachment at the meniscus, though the addition of HMW DNA reduces both biofilm attachment levels and biofilm strength. eDNA release events determine the sites for microcolony development [71] which might be mimicked by LMW DNA, leading to biofilm formation, and it is likely that the role of eDNA changes with biofilm development and the spatial heterogeneity in eDNA distributions and interactions with other matrix components add further complexity, as seen for *P. aeruginosa* PA01 [72]. In these biofilms, eDNA interacts with Pel and Psl EPS, forming cationic bridges to overcome electrostatic repulsion and allowing adhesion to surfaces, cell aggregation, and stabilizing the biofilm by forming a fiber-like network [9]. It is notable that eDNA is not restricted to S–L interface biofilms; it has been also associated with *P. aeruginosa* PA01 streamers [56] and clusters of planktonic cells [53] and therefore is not a biofilm-specific matrix component.

eDNA in bacterial biofilms is also known to promote the formation of amyloid fibers which are also critical for cell aggregation and attachment to surfaces. *P. fluorescens* SBW25 contains a Fap amyloid operon [25] and here we provide the first evidence using CLSM and AmyGreen [62,63] demonstrating that amyloid fibers are present in both Viscous Mass and Wrinkly Spreader biofilms, adding yet another layer of complexity to the matrix composition of these biofilms. Although our CLSM images suggest Fap fibers are present at equal levels in both biofilms, further work is required to confirm this using *fap* mutants which need to be produced for each of the wild-type and Wrinkly Spreader mutant strains, and to determine whether Fap levels change as the biofilms mature. As Fap fibers have been shown to increase cell aggregation and the surface hydrophobicity of *Pseudomonas* sp. UK4 cells [50,73] (Fap fibers are also produced by *P. aeruginosa* PA01, *P. fluorescens* Pf-5, and *P. putida* FI [25]), we might expect to find that Fap levels in the Wrinkly Spreader biofilm are higher, providing an explanation for the increased hydrophobic nature of Wrinkly Spreader cells and the dry nature of the top surface of Wrinkly Spreader biofilms. A direct comparison of *fap*, *pga*, and *wss* mutants in wild-type and Wrinkly Spreader backgrounds would also address the relative importance of Fap fibers, PNAG, and cellulose, respectively, in surface attachment and biofilm strength in this model system. *P. putida* KT2440 biofilms are likely to be even more complex, as this strain is also likely to produce Fap [25] and is known to produce alginate, cellulose, Pea and Peb EPS [35,38,39], as well as eDNA [34].

We have yet to start investigating the distribution of cellulose, eDNA, and Fap fibers in VM and WS biofilms, but expect differences in the distribution and quantity of matrix material, metabolically active and possibly motile cells, flagella, fimbriae, pili, LPS, etc., as well as lysing cells and cell debris, to contribute to spatial heterogeneity and larger-scale viscoelastic properties which differentiate Viscous Mass and Wrinkly Spreader biofilms [23]. It is clear from our DNase I treatments that eDNA has a greater role in early biofilm development, while our CLSM images suggest cellulose and eDNA aggregate into larger structures separate from the layer of producing cells. Earlier epifluorescent microscopy illustrated well the network of cellulose fibers seen in Viscous Mass and Wrinkly Spreader biofilms and encompassing large voids filled with bacteria [20,22,35]. Wild-type *P. fluorescens* SBW25 and Wrinkly Spreader mutant cells are capable of swimming motility and capable of aerotaxic migration that would allow them to colonize any part of the A–L interface biofilm [74], with fitness assays suggesting that the preferred Goldilocks’ zone for growth is at the top of the A–L interface [19] (CLSM suggests that the top-most layer of active cells is just below the exposed surface of the Wrinkly Spreader biofilm [52]). *P. fluorescens* SBW25 can utilize exogenous intermolecular agar networks and polyethylene glycol intermolecular friction to quickly colonize the A–L interface [74] suggesting that any viscous or viscoelastic matrix can be used as the basis for biofilm formation. It is likely therefore that cellulose and eDNA network structures or scaffolds might be colonized by motile cells to produce extensions from the original biofilm or new initial biofilm formation on uncolonized surfaces. *P. aeruginosa* PA01 eDNA-based planktonic cell clusters [53] may represent a surface-colonizing adaptation if survival and attachment rates are better than for single cells, in which case *P. fluorescens* SBW25 and other biofilm forming pseudomonads would also be expected to produce similar planktonic clusters.

There is also growing interest in other matrix components, such as membrane vesicles and proteins, which may play roles in biofilm structure and function [1,5,8,9,29,30,31,32]. The physically robust nature of the Wrinkly Spreader biofilm allowed us to readily separate cells and matrix material from the interstitial fluid, and we were able to visualize vesicles and demonstrate that eDNA was associated with these, as has been seen for *P. aeruginosa* PA01 [75,76]. *P. aeruginosa* PA01 membrane vesicles are produced by different mechanisms and have different compositions and functions [29,65,66,77]. We were able to demonstrate differences in protein profiles for biofilm matrix-associated proteins, biofilm vesicles and planktonic vesicles, which also suggest different origins and possible functions for these in the Wrinkly Spreader biofilm. We note that although membrane vesicles may diffuse away from cell surfaces, they spend more time at the liquid–bacterial membrane interface [78], raising the question of how significant their role might be in biofilms beyond regions with densely packed cells.

Although the underlying molecular biology of the *P. fluorescens* SBW25 Wrinkly Spreader biofilm was determined almost 20 years ago [18,19], we have felt that our understanding of this and the related Viscous Mass biofilm needed revision as progress has been made for other model systems where eDNA and amyloid fibers have been found to contribute to biofilm development and structure. Here, we demonstrate that eDNA and Fap amyloid fibers are also present in *P. fluorescens* SBW25 A–L interface biofilms, and eDNA plays a structural role in early development at least in the physically more robust Wrinkly Spreader biofilm. However, unlike *P. aeruginosa* S–L interface biofilms where eDNA is primarily responsible for the viscoelastic properties [69], eDNA plays a secondary role to cellulose which is the primary matrix and structural component of these *P. fluorescens* SBW25 biofilms.

## 4. Materials and Methods

### 4.1. Bacteria and Culturing Conditions

Wild-type *Pseudomonas fluorescens* SBW25 [24], which produces the Viscous Mass biofilm [22], and the archetypal Wrinkly Spreader (WS) mutant (*P. fluorescens* SBW25 *wspF* A901C) [21,79] and GFP-expressing WS-GFP strain (WS::miniTn7(Gm)_PAI/04/03_ *gfp*.ASV-a) [52], which produce the Wrinkly Spreader biofilm [24,79], were examined in this work. Microcosms were 30 mL glass vials containing 6 mL King’s B (KB) [80] liquid medium and supplemented with 1 mM FeCl_3_ to induce Viscous Mass biofilm-formation [22]. Bacteria were incubated at 20 °C with biofilms produced in microcosms incubated statically for 24 h or three days (for mature biofilms), and planktonic samples recovered from 24 h shaken microcosms in which biofilms could not form. Low-molecular-weight (LMW) salmon sperm DNA (Fluka, Charlotte, NC, USA) and high-molecular-weight (HMW) salmon testis DNA (Sigma-Aldrich, Dorset, UK) were added to microcosms at 100, 300, and 600 µg/mL, and Bovine pancreas deoxyribonuclease I (DNase I) (Sigma-Aldrich) added to 1 µg/mL to assess the importance of eDNA to biofilm development, final strength, and attachment levels.

### 4.2. Combined Biofilm Assay

Biofilms were quantitatively measured using the combined biofilm assay [57] based on earlier work [20,21,35] in microcosm vials which involves the serial measurement of biofilm strength (grams), attachment levels (crystal violet staining, A_570_), and total microcosm biomass (OD_600_) (i.e., growth). Biofilms were first assayed for strength using small (~0.01 g) glass balls which were sequentially added until the biofilm broke or sank (the maximum deformation or breaking mass [20,21,35]). It should be noted that although Viscous Mass biofilms developed in all treatments, confirmed by visual inspection, they were generally so weak that they could not easily be measured compared to the more robust Wrinkly Spreader biofilms [23]. The contents of the microcosm vials with damaged or sunken biofilms were transferred to a second vial which was then vigorously mixed for 30 s to disintegrate all biofilm material. This suspension was then sampled to determine optical density (OD_600_) using a Spectronic Helios Epsilon spectrophotometer (ThermoFisher Scientific, Oxford, UK) with 10 mm optical-path cuvettes, as a measure of total microcosm biomass. The original vials were then assayed for the attachment of cells and biofilm at the meniscus using crystal violet [20] (after [59], etc.). The vials were washed three times in water to remove non-attached cells and material, and then stained with 0.05% *w/v* crystal violet for 2 min and rewashed three times to remove unbound dye. The crystal violet was eluted with 5 mL ethanol and gentle shaking for 1 h and sampled to determine absorbance (A_570_) as a measure of biofilm attachment levels at the meniscus. A second elution with 5 mL 10% *v/v* acetic acid (after [61] etc.) for 1 h followed by absorbance (A_570_) measurements was used to quantify the remaining bound crystal violet.

### 4.3. Spectrophometric Measurement of eDNA

Quantitative measurements of eDNA levels in three-day-old biofilms were made by spectrometry [55]. Five hundred and fifty microliter samples were recovered from microcosms and the biofilms disrupted by vortexing for 10 min before cells and debris were pelleted by centrifugation at 12,000× *g* for 15 min and 500 µL of cell-free supernatant transferred to a new tube. DNA was precipitated with 50 µL 3 M sodium acetate (pH 5.2) and 1 mL 96% (*v*/*v*) ethanol at −20 °C overnight before centrifugation at 13,000× *g* for 15 min. The pelleted DNA was washed with 1 mL 70% (*v*/*v*) ethanol and subjected to centrifugation at 13,000× *g* for 15 min before being air-dried at room temperature. Samples were dissolved in 200 µL Tris-EDTA (TE) buffer and eDNA concentration measured using a NanoDrop 2000 spectrometer (ThermoFisher Scientific).

### 4.4. Confocal Laser Scanning Microscopy (CLSM) and Transmission Electron Microscopy (TEM)

Microscopy of biofilm samples was undertaken following established procedures [52]. Biofilm samples were recovered from three-day-old microcosms by spooling them onto P1000 pipettor tips and then rolling them out in the other direction onto microscope slides. Excess liquid was drained off before 10 µL aliquots of each of the required stains were added. eDNA was stained with 20 µM propidium iodide (Sigma-Aldrich) and 1 µM Experimental Stain 986 in DMSO (Department of Biomedicinal Chemistry, IMBG, Kyiv, Ukraine). Intracellular DNA from intact and metabolically active (live) cells was stained with 5 µM SYTO 9 solution (ThermoFisher Scientific), and total DNA (eDNA and DNA in active and dead cells) stained with 2 µg/mL ethidium bromide (ThermoFisher Scientific). Amyloid fibers were stained with 1 µM AmyGreen in DMSO (Department of Biomedicinal Chemistry), cellulose with 5 µM Calcofluor white (Sigma-Aldrich), and vesicle membranes with 5 µL Vybrant DiO solution (ThermoFisher Scientific). No additional washing was applied to the biofilm samples to minimize the physical disruption of biofilm structures through liquid movement. To stabilize vesicles, the suspension was mixed with molten 0.8% (*w*/*v*) agarose gel before applying it to the glass slide. Samples were not fixed, and a cover slip was put into position before imaging. CLSM analysis was undertaken using a Leica TCS SPE Confocal system with a coded DMi8 inverted microscope (Leica, Wetzlar, Germany) and Leica Application Suite X Version 3.4.1 software. Images were acquired using excitation at 488 nm and emission collected at 490–580 nm for AmyGreen, GFP, SYTO 9, and Vybrant DiO; excitation at 532 nm and emission collected at 537–670 for ethidium bromide, Experimental Stain 986, and propidium iodide; and excitation at 405 nm and emission collected at 450–500 nm for Calcofluor white.

Transmission electron microscopy (TEM) of biofilm samples was undertaken following standard procedures. Ten microliters of vesicle suspension were placed onto formvar-coated TEM grids and dried. Vesicles were stained with 2% (*w*/*v*) uranyl oxalate, pH 7.0, for 10 min and then washed with phosphate buffered saline (PBS). TEM was performed on a JEM 1230 (Jeol, Tokyo, Japan) microscope operated at 80 kV.

### 4.5. Membrane Vesicle (MV) Preparation

MVs were isolated from Wrinkly Spreader biofilms and planktonic cultures by centrifugation to remove cells and matrix followed by filtration (after [17] etc.). Two hundred milliliters of 24 h culture and three-day biofilms pooled from four 1 L flasks containing 250 mL KB medium were centrifuged at 8000× *g* at 4 °C for 20 min and the supernatant recovered. These were then centrifuged at 24,000× *g* 4 °C for 30 min and the supernatants passed through 0.45 µm pore-size PES filters (MDI, Gurgaon, India). The filtrates were pelleted by centrifugation at 60,000× *g* at 4 °C for 2 h and finally resuspended in 600 µL PBS. MV-associated proteins were separated by SDS-PAGE and visualized with PageBlue Protein Staining Solution (ThermoFisher Scientific). An aliquot of Pierce™ Unstained Protein MW Marker (ThermoFisher Scientific) was run on the gel for sizing. Samples were also treated with 100 µg/µL DNase I (Sigma-Aldrich) in 20 mM Tris-HCl pH7.4, 10 mM MgCl_2_, before the DNA was extracted with phenol-chloroform, precipitated with sodium acetate/ethanol as above, and finally resuspended in TE buffer. Samples were electrophoresed on a 1.3% (*w*/*v*) Tris-buffered EDTA (TBE)-agarose gel before being stained with ethidium bromide to visualize the DNA. An aliquot of O’GeneRuler 1 kb DNA ladder (ThermoFisher Scientific) was run on the gel for sizing.

### 4.6. Matrix-Associated Proteins

Matrix-associated proteins were recovered from Wrinkly Spreader biofilms after centrifugation to remove cells and extensive washing of the matrix material to remove unbound proteins. Five three-day biofilms grown in 6 mL KB microcosms were pooled, and the liquid carefully removed with a pipettor. Ten milliliters of deionized water were added and the biofilms gently mixed before leaving for 24 h. This washing was repeated a further six times before the biofilm matrix was pelleted by centrifugation at 4000× *g* for 10 min. The supernatant was carefully removed with a pipettor, and the matrix resuspended in the remaining liquid. 100 µL of biofilm matrix samples were pre-incubated with 50 µL deionized water, 0.1% (*w*/*v*) SDS, or 6 M urea at room temperature for 2 h before 20 µL SDS–PAGE sample buffer was added. Samples were investigated by SDS-PAGE as above.

### 4.7. MALDI-TOF Mass Spectometry and Protein Identification

Mass spectrometry of protein samples were undertaken following established procedures. Thirty microliters of membrane vesicle (MV) sample and matrix-associated protein were concentrated by SDS-PAGE to obtain lanes of 5 mm width. The lanes containing proteins were subjected to in-gel trypsinolysis and proteins identified by Maxis Impact LC-QTOF mass spectrometer (Bruker, Billerica, MA, USA) using the NCBInr database and Mascot server (Matrix Science, London, UK; http://www.matrixscience.com) to identify proteins and calculate exponentially modified protein abundance index (emPAI) values. Gene Ontology (GO) Annotation and protein subcellular locations were obtained from UniProt (www.uniprot.org) [81].

### 4.8. Statistical Analyses

Experiments were performed with replicates and means with standard errors (SE) are shown where appropriate. Statistical test and *p* values are rounded up to two decimal places, and significant results reported for α = 0.05 or when *p* ≤ 0.05. JMP Version 12 (SAS Institute Inc., Cary, NC, USA) statistical software was used to analyze data. First, residuals were investigated and the goodness of fit of a normal distribution determined using Shapiro–Wilk W test to decide whether to proceed with a parametric test or adopt a non-parametric approach. General linear mixed models (GLMM) were used to investigate combined biofilm assay data and determine significant effects and interactions, with LSMeans Differences Tukey HSD (α = 0.05) used to determine significant differences between means. *T*-tests and ANOVA models with post hoc Tukey–Kramer HSD tests (α = 0.05) were also used to make comparisons between means. Where appropriate and rather than to delete outliers, a non-parametric approach was followed using the Kruskal–Wallis (Rank Sums) test and comparisons of each mean made using the Wilcoxon method. Spearman’s Rho was used to determine correlations. Contingency tables and χ^2^ tests were used to assess the independence of *protein source* × *abundance* and *protein source* × *cellular localization* using the Chi-Square Calculator (www.icalcu.com/stat/chisqtest.html, last accessed on 30 November 2022).

## Data Availability

All data have been included in this manuscript (figures).

## Supplementary Materials

The following supporting information can be downloaded at: https://www.mdpi.com/article/10.3390/ijms232315096/s1.
