# Supplementary material for: eDNA, Amyloid Fibers and Membrane Vesicles Identified in Pseudomonas fluorescens SBW25 Biofilms
Source: Int J Mol Sci. 2022 Dec 1;23(23):15096. doi: 10.3390/ijms232315096 (PMC9738004; doi:10.3390/ijms232315096)
Supplement: Supplementary file 1 [file ijms-23-15096-s001.zip › Figure S1.pdf]

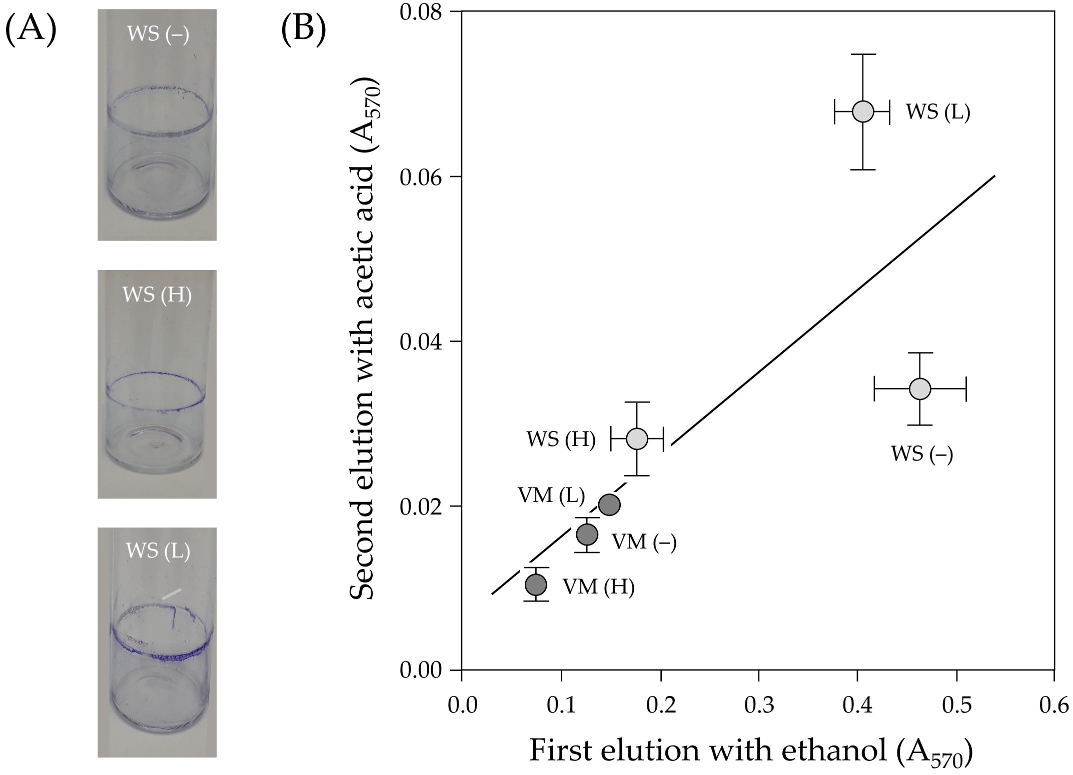

Figure S1. LMW DNA enhances Crystal violet staining of the meniscus region in Wrinkly Spreader microcosms. Shown here (**A**) are 3 day-old Wrinkly Spreader microcosms which have been stained with Crystal violet to indicate cells adhered to the vial walls at the meniscus, and (**B**) absorbance (A_{570}) measurements of Crystal violet eluted first by ethanol (*x*-axis) and then by acetic acid (*y*-axis). Means (n = 8 - 24) ± SE are shown. See the main text for statistical differences. The diagonal line indicates the correlation between first and second elutions (Spearman's Rho = 0.78).

Moshynets *et al.*
