# Supplementary material for: eDNA, Amyloid Fibers and Membrane Vesicles Identified in Pseudomonas fluorescens SBW25 Biofilms
Source: Int J Mol Sci. 2022 Dec 1;23(23):15096. doi: 10.3390/ijms232315096 (PMC9738004; doi:10.3390/ijms232315096)
Supplement: Supplementary file 1 [file ijms-23-15096-s001.zip › Figure S2.pdf]

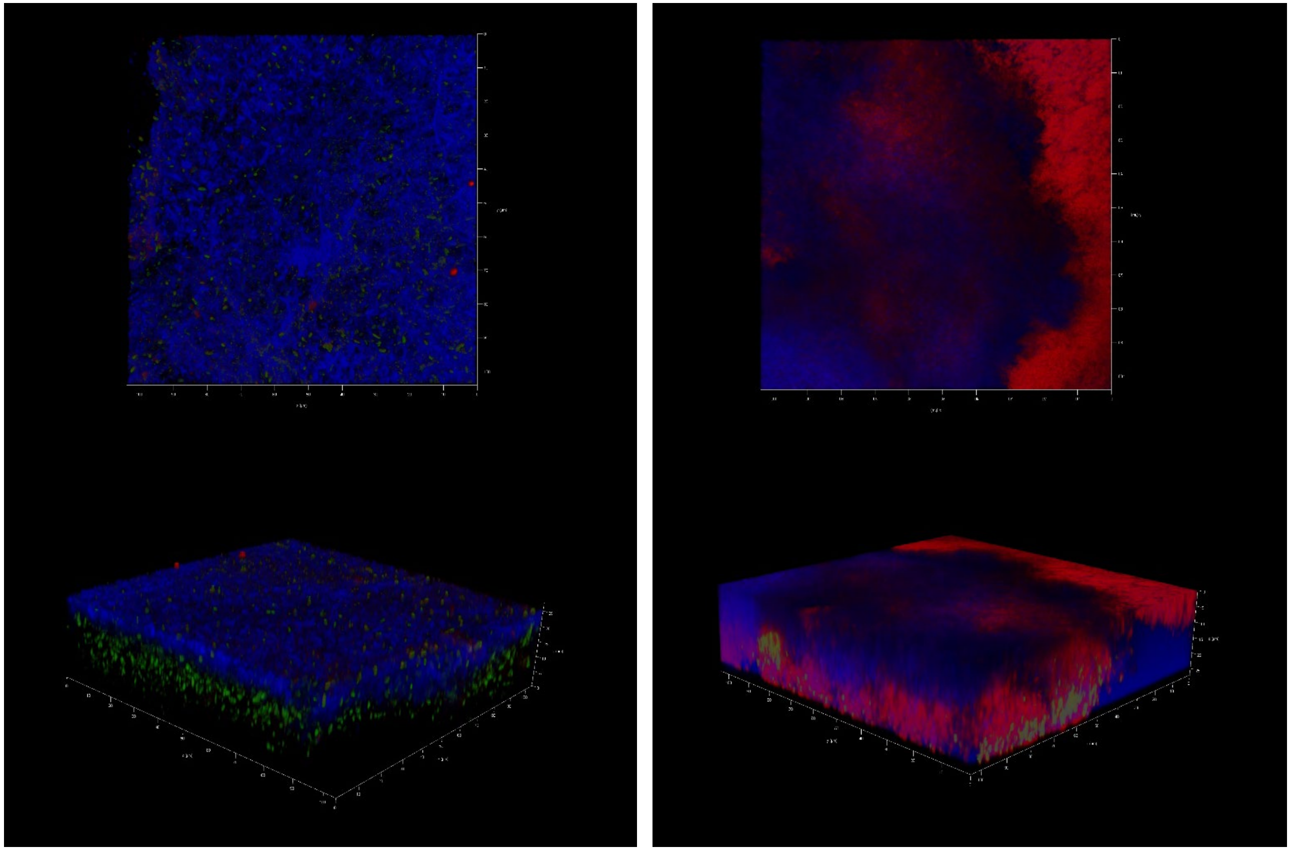

Figure S2. Experimental Stain 896 used to visualize eDNA. A preliminary comparison of eDNA staining by propidium iodide and Experimental Stain 986 was undertaken by CLSM with three-day old Wrinkly Spreader biofilm samples incubated with 300 ug/ml LMW DNA. CYTO9 was used to visualize intracellular DNA of live cells and Calcofluor white to visualize cellulose. Shown here are combined images with (left) propidium iodide (red channel), SYTO9 (green channel) and Calcofluor white (blue channel), and (right) Experimental Stain 986 (red channel), SYTO9 (green channel) and Calcofluor white (blue channel). The images are 105 x 105 x 25/20 µm.
