# Supplementary material for: eDNA, Amyloid Fibers and Membrane Vesicles Identified in Pseudomonas fluorescens SBW25 Biofilms
Source: Int J Mol Sci. 2022 Dec 1;23(23):15096. doi: 10.3390/ijms232315096 (PMC9738004; doi:10.3390/ijms232315096)
Supplement: Supplementary file 1 [file ijms-23-15096-s001.zip › Figure S3.pdf]

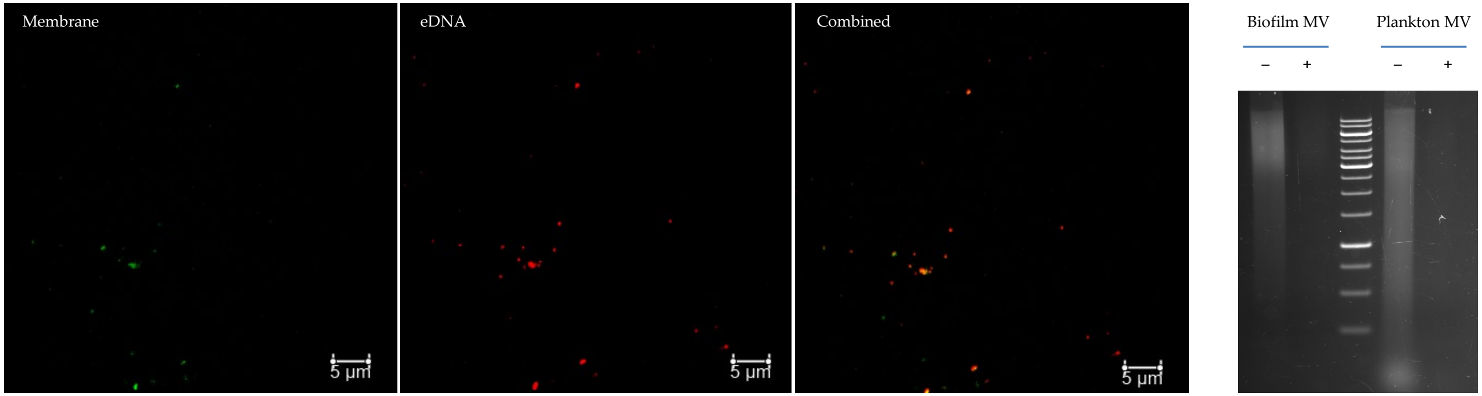

Figure S3. eDNA is associated with membrane vesicles. Membrane vesicles (MVs) were isolated from Wrinkly Spreader biofilms and planktonic samples and investigated by CLSM and DNA electrophoresis. Shown here (left) are single-channel and composite images of a biofilm sample with Vybrant DiO (green channel) and ethidium bromide (red channel) used to visualize MVs and eDNA, respectively. The scale bars indicate 5 µm. Also shown (right) are biofilm and planktonic MV samples pre-treated with DNase 1 (+) before electrophoresis with an aliquot of O'GeneRuler 1 kb DNA ladder run in the central lane for reference.
