# Supplementary material for: eDNA, Amyloid Fibers and Membrane Vesicles Identified in Pseudomonas fluorescens SBW25 Biofilms
Source: Int J Mol Sci. 2022 Dec 1;23(23):15096. doi: 10.3390/ijms232315096 (PMC9738004; doi:10.3390/ijms232315096)
Supplement: Supplementary file 1 [file ijms-23-15096-s001.zip › Figure S4.pdf]

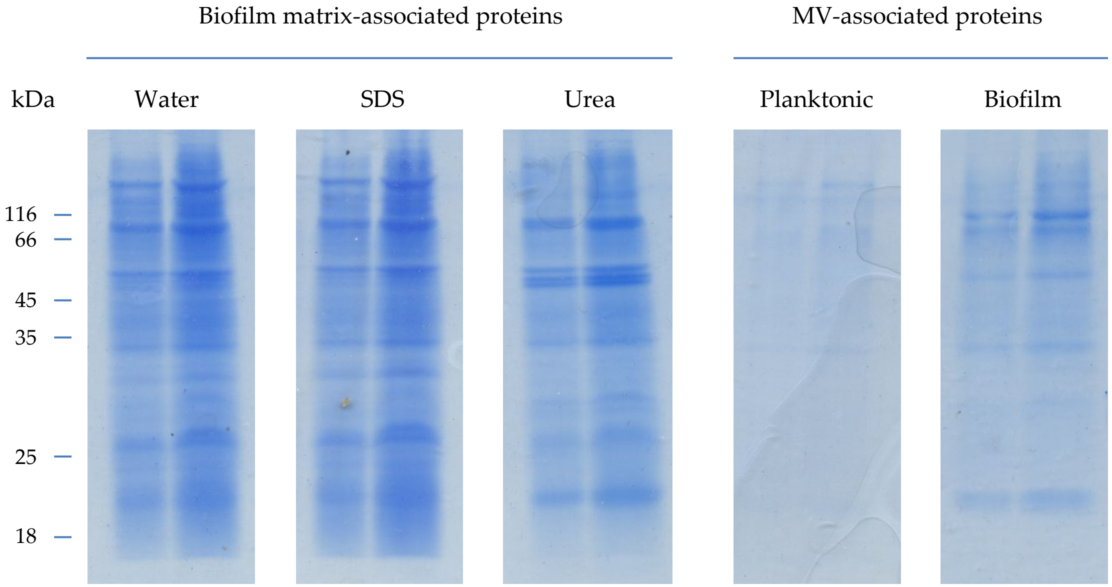

Figure S4. Wrinkly Spreader biofilm matrix and membrane vesicle-associated proteins. Biofilm matrix-associated proteins are retained in biofilm samples despite extensive washing, and membrane vesicles (MVs) can be isolated from cells and other material by a combination of centrifugation and filtration of biofilm and planktonic (shaken) culture samples. Shown here are proteins separated by denaturing 13% SDS-PAGE and visualized with PageBlue. Biofilm matrix-associated proteins were pre-incubated in deionized water, SDS or urea before SDS-PAGE buffer was added. Five and ten microliters of each sample were loaded onto the gel and the relative staining of each panel reflects the difference in total protein recovered in each treatment. Protein molecular weight marker was used to determine kDa.
