# Supplementary material for: eDNA, Amyloid Fibers and Membrane Vesicles Identified in Pseudomonas fluorescens SBW25 Biofilms
Source: Int J Mol Sci. 2022 Dec 1;23(23):15096. doi: 10.3390/ijms232315096 (PMC9738004; doi:10.3390/ijms232315096)
Supplement: Supplementary file 1 [file ijms-23-15096-s001.zip › Table S1.pdf]

Table S1: **GLM details and effects tests**

Effects	NParm	DF	Response variables: Microcosm Biomass	Biofilm Strength	Biofilm Attachment
			P Value	*P* Value	*P* Value
Biofilm type (Viscous Mass & Wrinkly Spreader)	1	1	< 0.01	< 0.01	< 0.01
DNA treatment (Ctrl, LMW, HMW)	2	2	< 0.01	< 0.01	< 0.01
DNA concentration (0, 100, 300 & 600 µg/ml)	1	1	0.58	0.92	0.21
Replicate (1 - 8)	7	7	0.10	0.38	0.03*
Biofilm type x *DNA treatment* interaction	2	2	< 0.01	< 0.01	< 0.01

Three separate GLM models were run with microcosm biomass (growth), biofilm strength and biofilm attachment as Response variables. *Biofilm type* (categorical variable), *DNA treatment* (categorical variable), *DNA concentration* (continuous variable) and Replicate (categorical variable) as effects, and *Biofilm type x DNA treatment* as an interaction (note that *Replicate* could not be modelled as a random effect because model residuals were not Normally distributed). Model summaries (all statistics rounded up to two decimal places): Microcosm biomass (OD_{600}), $r^2 = 0.77$; ANOVA, $F_{13,93} = 23.40$, $P < 0.01$; Biofilm strength (MDM, g), $r^2 = 0.86$; ANOVA, $F_{13,93} = 44.81$, $P < 0.01$; Biofilm attachment (A_{570}), $r^2 = 0.72$; ANOVA, $F_{13,93} = 17.98$, $P < 0.01$. NParm, Number of parameters; DF, Degrees of Freedom.
* Effect further examined by LSMeans Differences Tukey HSD and no significant difference between means was found ($Q = 3.10$, $\alpha = 0.05$).
