# Supplementary material for: eDNA, Amyloid Fibers and Membrane Vesicles Identified in Pseudomonas fluorescens SBW25 Biofilms
Source: Int J Mol Sci. 2022 Dec 1;23(23):15096. doi: 10.3390/ijms232315096 (PMC9738004; doi:10.3390/ijms232315096)
Supplement: Supplementary file 1 [file ijms-23-15096-s001.zip › Table S2.pdf]

Biofilm matrix sample

Note: Human contaminants removed.

ID	Protein	prot_score	emPAI
gi|229364771|emb|CAY52770.1|	elongation factor TU [Pseudomonas fluorescens SBW25]	11287	10.9
gi|229364759|emb|CAY52749.1|	50S ribosomal protein L14 [Pseudomonas fluorescens SBW25]	650	9.09
gi|229364173|emb|CAY51832.1|	putative ferritin DPS-family DNA binding protein [Pseudomonas fluorescens SBW25]	2142	6.17
gi|229364240|emb|CAY51939.1|	chaperone Hsp60, peptide-dependent ATPase, heat shock protein [Pseudomonas fluorescens SBW25]	9591	4.53
gi|229360727|emb|CAY47585.1|	putative membrane protein [Pseudomonas fluorescens SBW25]	1059	3.64
gi|229361881|emb|CAY48781.1|	ferripyoverdine receptor precursor [Pseudomonas fluorescens SBW25]	13476	3.15
gi|229365347|emb|CAY53725.1|	ATP synthase beta chain [Pseudomonas fluorescens SBW25]	4471	2.7
gi|229361202|emb|CAY48066.1|	succinate dehydrogenase iron-sulfur protein [Pseudomonas fluorescens SBW25]	1915	2.4
gi|229364757|emb|CAY52745.1|	50S ribosomal protein L5 [Pseudomonas fluorescens SBW25]	659	2.32
gi|229364222|emb|CAY51910.1|	haem utilisation protein [Pseudomonas fluorescens SBW25]	496	2.11
gi|229364824|emb|CAY52855.1|	conserved hypothetical protein [Pseudomonas fluorescens SBW25]	3803	2.09
gi|229359711|emb|CAY46559.1|	conserved hypothetical protein [Pseudomonas fluorescens SBW25]	424	2.08
gi|229365218|emb|CAY53512.1|	acetylglutamate kinase [Pseudomonas fluorescens SBW25]	1343	2.05
gi|229363881|emb|CAY51363.1|	putative outer membrane porin A protein [Pseudomonas fluorescens SBW25]	7857	1.82
gi|229363442|emb|CAY50639.1|	50S ribosomal protein L20 [Pseudomonas fluorescens SBW25]	49	1.82
gi|229364725|emb|CAY52696.1|	putative ABC transport system, outer membrane exported protein [Pseudomonas fluorescens SBW25]	435	1.74
gi|229361883|emb|CAY48783.1|	putative pyoverdine synthetase F [Pseudomonas fluorescens SBW25]	2743	1.72
gi|229364778|emb|CAY52781.1|	50S ribosomal protein L10 [Pseudomonas fluorescens SBW25]	1487	1.69
gi|229364221|emb|CAY51907.1|	putative exported heme receptor protein [Pseudomonas fluorescens SBW25]	8771	1.64
gi|229364763|emb|CAY52756.1|	30S ribosomal protein S3 [Pseudomonas fluorescens SBW25]	343	1.62
gi|229360144|emb|CAY47001.1|	ribose-phosphate pyrophosphokinase [Pseudomonas fluorescens SBW25]	1083	1.56
gi|229360670|emb|CAY47528.1|	elongation factor [Pseudomonas fluorescens SBW25]	433	1.56
gi|229364311|emb|CAY52052.1|	nucleoside diphosphate kinase [Pseudomonas fluorescens SBW25]	848	1.54
gi|229363277|emb|CAY50379.1|	outer membrane efflux protein [Pseudomonas fluorescens SBW25]	1225	1.53
gi|229365349|emb|CAY53727.1|	ATP synthase alpha chain [Pseudomonas fluorescens SBW25]	3739	1.49
gi|229362497|emb|CAY49404.1|	conserved hypothetical protein [Pseudomonas fluorescens SBW25]	859	1.49
gi|229360462|emb|CAY47319.1|	cytosol aminopeptidase [Pseudomonas fluorescens SBW25]	3238	1.45
gi|229363728|emb|CAY51128.1|	flagellin [Pseudomonas fluorescens SBW25]	3882	1.43
gi|229365095|emb|CAY53304.1|	putative outer membrane protein [Pseudomonas fluorescens SBW25]	2004	1.41
gi|229364744|emb|CAY52723.1|	DNA-directed RNA polymerase alpha chain [Pseudomonas fluorescens SBW25]	1181	1.41
gi|229364790|emb|CAY52800.1|	N-acetyl-gamma-glutamyl-phosphate reductase [Pseudomonas fluorescens SBW25]	794	1.41
gi|229359883|emb|CAY46736.1|	pyruvate dehydrogenase E1 component [Pseudomonas fluorescens SBW25]	3865	1.4
gi|229365134|emb|CAY53370.1|	thioredoxin [Pseudomonas fluorescens SBW25]	163	1.39
gi|229363660|emb|CAY51010.1|	diaminobutyrate--2-oxoglutarate aminotransferase [Pseudomonas fluorescens SBW25]	2149	1.38
gi|229364145|emb|CAY51782.1|	ornithine carbamoyltransferase, catabolic [Pseudomonas fluorescens SBW25]	1382	1.34
gi|229364712|emb|CAY52676.1|	6,7-dimethyl-8-ribityllumazine synthase [Pseudomonas fluorescens SBW25]	892	1.34

gi|229365209|emb|CAY53495.1|	putative aldehyde dehydrogenase [Pseudomonas fluorescens SBW25]	604	1.27
gi|229362083|emb|CAY48985.1|	putative short chain dehydrogenase [Pseudomonas fluorescens SBW25]	291	1.25
gi|229364752|emb|CAY52736.1|	30S ribosomal protein S5 [Pseudomonas fluorescens SBW25]	179	1.21
gi|229361551|emb|CAY48427.1|	two-component system, response regulator [Pseudomonas fluorescens SBW25]	121	1.15
gi|229365227|emb|CAY53528.1|	conserved hypothetical protein [Pseudomonas fluorescens SBW25]	370	1.15
gi|229364746|emb|CAY52727.1|	30S ribosomal protein S11 [Pseudomonas fluorescens SBW25]	64	1.14
gi|229361036|emb|CAY47898.1|	30S ribosomal protein S1 [Pseudomonas fluorescens SBW25]	1286	1.13
gi|229359682|emb|CAY46530.1|	putative oxidoreductase [Pseudomonas fluorescens SBW25]	477	1.12
gi|229363225|emb|CAY50308.1|	DNA-binding protein HU-beta [Pseudomonas fluorescens SBW25]	334	1.09
gi|229363273|emb|CAY50374.1|	putative L-ornithine 5-monooxygenase [Pseudomonas fluorescens SBW25]	2964	1.08
gi|229364779|emb|CAY52783.1|	50S ribosomal protein L1 [Pseudomonas fluorescens SBW25]	737	1.08
gi|229363652|emb|CAY50995.1|	cell division inhibitor [Pseudomonas fluorescens SBW25]	647	1.08
gi|229365256|emb|CAY53574.1|	chaperone [Pseudomonas fluorescens SBW25]	1289	1.04
gi|229359882|emb|CAY46735.1|	dihydrolipoyllysine-residue acetyltransferase component of pyruvate dehydrogenase complex [Pseudomonas fluorescens SB	3298	1.03
gi|229360251|emb|CAY47108.1|	30S ribosomal protein S9 [Pseudomonas fluorescens SBW25]	95	1.03
gi|229364468|emb|CAY52297.1|	ketol-acid reductoisomerase [Pseudomonas fluorescens SBW25]	1754	1
gi|229360318|emb|CAY47175.1|	putative lipoprotein [Pseudomonas fluorescens SBW25]	2746	0.98
gi|229364964|emb|CAY53084.1|	putative exported protein [Pseudomonas fluorescens SBW25]	603	0.98
gi|229359943|emb|CAY46797.1|	putative phage-related protein [Pseudomonas fluorescens SBW25]	998	0.94
gi|229360872|emb|CAY47731.1|	phosphoribosylaminoimidazole-succinocarboxamide synthase [Pseudomonas fluorescens SBW25]	698	0.94
gi|229360669|emb|CAY47527.1|	30S ribosomal protein S2 [Pseudomonas fluorescens SBW25]	798	0.93
gi|229360143|emb|CAY47000.1|	putative ribosomal protein L25p family [Pseudomonas fluorescens SBW25]	240	0.92
gi|229359944|emb|CAY46798.1|	putative phage-related protein [Pseudomonas fluorescens SBW25]	333	0.91
gi|229359623|emb|CAY46465.1|	4-aminobutyrate aminotransferase [Pseudomonas fluorescens SBW25]	1342	0.9
gi|229360243|emb|CAY47100.1|	putative membrane protein [Pseudomonas fluorescens SBW25]	102	0.9
gi|229364385|emb|CAY52165.1|	putative aminotransferase [Pseudomonas fluorescens SBW25]	334	0.89
gi|229360012|emb|CAY46866.1|	putative outer membrane protein [Pseudomonas fluorescens SBW25]	785	0.88
gi|229363109|emb|CAY50118.1|	isocitrate dehydrogenase [NADP] [Pseudomonas fluorescens SBW25]	1675	0.88
gi|229359622|emb|CAY46464.1|	succinate-semialdehyde dehydrogenase [NADP+] [Pseudomonas fluorescens SBW25]	909	0.87
gi|229359816|emb|CAY46666.1|	ubiquinone/menaquinone biosynthesis methyltransferase [Pseudomonas fluorescens SBW25]	108	0.87
gi|229360534|emb|CAY47391.1|	putative aminotransferase [Pseudomonas fluorescens SBW25]	1236	0.86
gi|229364178|emb|CAY51840.1|	putative family S43 non-peptidase homologue [Pseudomonas fluorescens SBW25]	1171	0.85
gi|229363994|emb|CAY51541.1|	ribonucleoside-diphosphate reductase alpha chain [Pseudomonas fluorescens SBW25]	2773	0.84
gi|229364772|emb|CAY52772.1|	elongation factor G [Pseudomonas fluorescens SBW25]	2159	0.84
gi|229360305|emb|CAY47162.1|	ATP phosphoribosyltransferase [Pseudomonas fluorescens SBW25]	159	0.84
gi|260594103|gb|ACX46346.1|	RpoB, partial [Pseudomonas fluorescens SBW25]	351	0.83
gi|229363228|emb|CAY50311.1|	ATP-dependent Clp protease proteolytic subunit ClpP [Pseudomonas fluorescens SBW25]	480	0.83
gi|229360601|emb|CAY47458.1|	ferredoxin--NADP reductase [Pseudomonas fluorescens SBW25]	93	0.82
gi|229361207|emb|CAY48071.1|	succinyl-CoA synthetase alpha chain [Pseudomonas fluorescens SBW25]	181	0.81
gi|229364160|emb|CAY51808.1|	putative OmpA family lipoprotein [Pseudomonas fluorescens SBW25]	3164	0.81

gi|229363110|emb|CAY50120.1|	isocitrate dehydrogenase [NADP] [Pseudomonas fluorescens SBW25]	1968	0.8
gi|229364573|emb|CAY52457.1|	conserved hypothetical ABC transport, membrane protein [Pseudomonas fluorescens SBW25]	927	0.8
gi|229363689|emb|CAY51060.1|	chemotaxis protein CheW [Pseudomonas fluorescens SBW25]	77	0.8
gi|229364498|emb|CAY52345.1|	polynucleotide phosphorylase/polyadenylase [Pseudomonas fluorescens SBW25]	3009	0.79
gi|229362326|emb|CAY49228.1|	putative aldo/keto reductase [Pseudomonas fluorescens SBW25]	788	0.79
gi|229361205|emb|CAY48069.1|	dihydrolipoyl dehydrogenase [Pseudomonas fluorescens SBW25]	518	0.78
gi|229360869|emb|CAY47728.1|	dihydrodipicolinate synthase [Pseudomonas fluorescens SBW25]	561	0.77
gi|229365250|emb|CAY53564.1|	conserved hypothetical protein [Pseudomonas fluorescens SBW25]	774	0.77
gi|229363172|emb|CAY50218.1|	peptidyl-prolyl cis-trans isomerase B [Pseudomonas fluorescens SBW25]	393	0.77
gi|229365348|emb|CAY53726.1|	ATP synthase gamma chain [Pseudomonas fluorescens SBW25]	469	0.76
gi|229363264|emb|CAY50358.1|	lipoamide acyltransferase component of branched-chain alpha-keto acid dehydrogenase complex [Pseudomonas fluorescens	564	0.75
gi|229363458|emb|CAY50667.1|	putative L-asparaginase II precursor [Pseudomonas fluorescens SBW25]	538	0.74
gi|229361138|emb|CAY48001.1|	molybdenum cofactor biosynthesis protein B [Pseudomonas fluorescens SBW25]	404	0.74
gi|229360153|emb|CAY47010.1|	putative exported protein [Pseudomonas fluorescens SBW25]	101	0.74
gi|229360774|emb|CAY47632.1|	outer membrane protein precursor [Pseudomonas fluorescens SBW25]	955	0.73
gi|229360293|emb|CAY47150.1|	putative ABC transport system, ATP-binding protein [Pseudomonas fluorescens SBW25]	118	0.71
gi|229364518|emb|CAY52373.1|	chaperone protein DnkA (HSP70) [Pseudomonas fluorescens SBW25]	2267	0.7
gi|229360973|emb|CAY47833.1|	transaldolase B [Pseudomonas fluorescens SBW25]	187	0.7
gi|229363895|emb|CAY51388.1|	putative alanyl-tRNA synthetase [Pseudomonas fluorescens SBW25]	316	0.7
gi|229364775|emb|CAY52776.1|	DNA-directed RNA polymerase beta~ chain [Pseudomonas fluorescens SBW25]	3147	0.68
gi|229360548|emb|CAY47405.1|	glycerol kinase [Pseudomonas fluorescens SBW25]	1701	0.68
gi|229365251|emb|CAY53566.1|	conserved hypothetical protein [Pseudomonas fluorescens SBW25]	1735	0.68
gi|229363229|emb|CAY50312.1|	trigger factor [Pseudomonas fluorescens SBW25]	759	0.68
gi|229362316|emb|CAY49218.1|	alkyl hydroperoxide reductase protein [Pseudomonas fluorescens SBW25]	506	0.68
gi|229363888|emb|CAY51376.1|	putative phosphoenolpyruvate synthase [Pseudomonas fluorescens SBW25]	2008	0.66
gi|229363697|emb|CAY51074.1|	chemotaxis protein CheY [Pseudomonas fluorescens SBW25]	169	0.65
gi|229364995|emb|CAY53133.1|	aspartate carbamoyltransferase [Pseudomonas fluorescens SBW25]	309	0.64
gi|229360254|emb|CAY47111.1|	putative ubiquinol--cytochrome C reductase, cytochrome C1 [Pseudomonas fluorescens SBW25]	180	0.63
gi|229361880|emb|CAY48780.1|	pvdj(2) [Pseudomonas fluorescens SBW25]	7522	0.62
gi|229364817|emb|CAY52842.1|	putative exported organic solvent tolerance protein [Pseudomonas fluorescens SBW25]	521	0.62
gi|229360360|emb|CAY47217.1|	cell division protein [Pseudomonas fluorescens SBW25]	336	0.62
gi|229360557|emb|CAY47414.1|	putative alkyl hydroperoxide reductase [Pseudomonas fluorescens SBW25]	495	0.62
gi|229363782|emb|CAY51208.1|	GTP pyrophosphokinase [Pseudomonas fluorescens SBW25]	429	0.61
gi|229360249|emb|CAY47106.1|	NADP(H)-dependt aldo-keto reductase [Pseudomonas fluorescens SBW25]	346	0.59
gi|229364776|emb|CAY52777.1|	DNA-directed RNA polymerase beta chain [Pseudomonas fluorescens SBW25]	1409	0.58
gi|229362693|emb|CAY49603.1|	putative ferric alcaligin siderophore receptor [Pseudomonas fluorescens SBW25]	1899	0.58
gi|229361201|emb|CAY48065.1|	succinate dehydrogenase flavoprotein subunit [Pseudomonas fluorescens SBW25]	1979	0.58
gi|229359726|emb|CAY46574.1|	outer membrane protein, oprE3 precursor [Pseudomonas fluorescens SBW25]	1656	0.58
gi|229364115|emb|CAY51736.1|	putative exported protein [Pseudomonas fluorescens SBW25]	1185	0.58
gi|229362111|emb|CAY49013.1|	putative ribonucleoside-diphosphate reductase beta chain [Pseudomonas fluorescens SBW25]	178	0.58

gi|229364032|emb|CAY51597.1|	ribonucleoside-diphosphate reductase beta chain [Pseudomonas fluorescens SBW25]	161	0.58
gi|229364521|emb|CAY52377.1|	ferric uptake regulation protein [Pseudomonas fluorescens SBW25]	167	0.58
gi|229360742|emb|CAY47600.1|	branched amino acid ABC transport system, substrate-binding protein [Pseudomonas fluorescens SBW25]	503	0.57
gi|229364415|emb|CAY52212.1|	putative putative oxidoreduxtase [Pseudomonas fluorescens SBW25]	210	0.56
gi|229360807|emb|CAY47665.1|	probable alcohol dehydrogenase [Pseudomonas fluorescens SBW25]	228	0.56
gi|229360927|emb|CAY47787.1|	putative ATP-binding protein [Pseudomonas fluorescens SBW25]	218	0.56
gi|229362018|emb|CAY48919.1|	putative ferric alcaligin siderophore receptor [Pseudomonas fluorescens SBW25]	559	0.55
gi|229364293|emb|CAY52028.1|	GMP synthase (glutamine-hydrolyzing) [Pseudomonas fluorescens SBW25]	250	0.54
gi|229361204|emb|CAY48068.1|	dihydrolipoyllysine-residue succinyltransferase component of 2-oxoglutarate dehydrogenase complex [Pseudomonas fluore	703	0.53
gi|229362920|emb|CAY49837.1|	L-lactate dehydrogenase [Pseudomonas fluorescens SBW25]	108	0.53
gi|229365230|emb|CAY53532.1|	biopolymer transport membrane protein [Pseudomonas fluorescens SBW25]	1740	0.53
gi|229361882|emb|CAY48782.1|	putative ABC transporter ATP-binding protein [Pseudomonas fluorescens SBW25]	734	0.52
gi|229361046|emb|CAY47908.1|	UDP-N-acetylglucosamine2-epimerase [Pseudomonas fluorescens SBW25]	113	0.52
gi|229364164|emb|CAY51814.1|	TolQ protein [Pseudomonas fluorescens SBW25]	397	0.52
gi|229363585|emb|CAY50877.1|	putative putative heat shock protein A [Pseudomonas fluorescens SBW25]	192	0.52
gi|229360685|emb|CAY47543.1|	acetyl-coenzyme A carboxylase carboxyl transferase subunit alpha [Pseudomonas fluorescens SBW25]	122	0.51
gi|229361213|emb|CAY48077.1|	chaperone (heat shock protein) [Pseudomonas fluorescens SBW25]	322	0.5
gi|229360955|emb|CAY47815.1|	glyceraldehyde-3-phosphate dehydrogenase [Pseudomonas fluorescens SBW25]	278	0.5
gi|229364318|emb|CAY52065.1|	cysteine desulfurase [Pseudomonas fluorescens SBW25]	639	0.5
gi|229363945|emb|CAY51470.1|	electron transfer flavoprotein beta-subunit [Pseudomonas fluorescens SBW25]	365	0.5
gi|229364503|emb|CAY52353.1|	transcription termination factor [Pseudomonas fluorescens SBW25]	900	0.49
gi|229364218|emb|CAY51902.1|	glyceraldehyde 3-phosphate dehydrogenase 1 [Pseudomonas fluorescens SBW25]	951	0.49
gi|229361050|emb|CAY47913.1|	putative dehydrogenase [Pseudomonas fluorescens SBW25]	126	0.49
gi|229363756|emb|CAY51170.1|	putative fatty acid transport system, membrane protein [Pseudomonas fluorescens SBW25]	427	0.48
gi|229360407|emb|CAY47264.1|	putative amino acid ABC transport system, substrate-binding protein [Pseudomonas fluorescens SBW25]	94	0.48
gi|229363928|emb|CAY51444.1|	putative amino transferase [Pseudomonas fluorescens SBW25]	912	0.48
gi|229359974|emb|CAY46828.1|	putative amino acid oxidase [Pseudomonas fluorescens SBW25]	572	0.48
gi|229362537|emb|CAY49444.1|	putative TonB-dependent outer membrane receptor [Pseudomonas fluorescens SBW25]	1147	0.47
gi|229364144|emb|CAY51781.1|	arginine deiminase [Pseudomonas fluorescens SBW25]	1132	0.47
gi|229365133|emb|CAY53368.1|	transcription termination factor [Pseudomonas fluorescens SBW25]	230	0.47
gi|229363266|emb|CAY50361.1|	putative branched-chain-amino-acid aminotransferase [Pseudomonas fluorescens SBW25]	226	0.47
gi|229360544|emb|CAY47401.1|	glutamate/aspartate ABC transport system, permease [Pseudomonas fluorescens SBW25]	228	0.47
gi|229362357|emb|CAY49259.1|	putative phenylacetic acid degradation enoyl-CoA hydratase [Pseudomonas fluorescens SBW25]	593	0.47
gi|229360942|emb|CAY47802.1|	fatty oxidation complex alpha subunit [Pseudomonas fluorescens SBW25]	624	0.46
gi|229364474|emb|CAY52307.1|	conserved hypothetical protein [Pseudomonas fluorescens SBW25]	426	0.46
gi|229360546|emb|CAY47403.1|	aerobic glycerol-3-phosphate dehydrogenase [Pseudomonas fluorescens SBW25]	208	0.46
gi|229360593|emb|CAY47450.1|	recombinase A [Pseudomonas fluorescens SBW25]	499	0.46
gi|229360496|emb|CAY47353.1|	putative iron utilisation protein [Pseudomonas fluorescens SBW25]	407	0.46
gi|229364029|emb|CAY51593.1|	putative histidine-binding periplasmic protein precursor [Pseudomonas fluorescens SBW25]	80	0.46
gi|229365246|emb|CAY53558.1|	conserved hypothetical protein [Pseudomonas fluorescens SBW25]	163	0.45

gi|229360089|emb|CAY46943.1|	omega-amino acid--pyruvate aminotransferase [Pseudomonas fluorescens SBW25]	99	0.45
gi|229359677|emb|CAY46525.1|	putative ABC transport system, exported protein [Pseudomonas fluorescens SBW25]	485	0.45
gi|229365081|emb|CAY53280.1|	putrescine ABC transport system, substrate-binding periplasmic protein precursor [Pseudomonas fluorescens SBW25]	336	0.44
gi|229360687|emb|CAY47545.1|	CTP synthase [Pseudomonas fluorescens SBW25]	196	0.44
gi|229364186|emb|CAY51851.1|	preprotein translocase, ATPase secretion component (General Secretory Pathway) [Pseudomonas fluorescens SBW25]	1047	0.43
gi|229364150|emb|CAY51790.1|	glycine dehydrogenase [decarboxylating] [Pseudomonas fluorescens SBW25]	661	0.43
gi|229360333|emb|CAY47190.1|	putative exported protein [Pseudomonas fluorescens SBW25]	284	0.43
gi|229365217|emb|CAY53511.1|	putative phosphomannomutase/phosphoglucomutase [Pseudomonas fluorescens SBW25]	145	0.43
gi|229364766|emb|CAY52761.1|	50S ribosomal protein L2 [Pseudomonas fluorescens SBW25]	180	0.43
gi|229363699|emb|CAY51077.1|	ParA family ATPase [Pseudomonas fluorescens SBW25]	90	0.43
gi|229361985|emb|CAY48885.1|	conserved hypothetical protein [Pseudomonas fluorescens SBW25]	386	0.43
gi|229361879|emb|CAY48779.1|	pyoverdine synthetase I [Pseudomonas fluorescens SBW25]	7077	0.42
gi|229364959|emb|CAY53075.1|	adenosylhomocysteinase [Pseudomonas fluorescens SBW25]	853	0.42
gi|229365169|emb|CAY53427.1|	argininosuccinate lyase [Pseudomonas fluorescens SBW25]	629	0.42
gi|229359775|emb|CAY46625.1|	glutamine synthetase [Pseudomonas fluorescens SBW25]	1068	0.42
gi|229361206|emb|CAY48070.1|	succinyl-CoA synthetase beta chain [Pseudomonas fluorescens SBW25]	434	0.42
gi|229364257|emb|CAY51969.1|	threonine synthase [Pseudomonas fluorescens SBW25]	306	0.42
gi|229362358|emb|CAY49260.1|	acyl-CoA dehydrogenase [Pseudomonas fluorescens SBW25]	163	0.42
gi|229364179|emb|CAY51842.1|	putative transmembrane protein [Pseudomonas fluorescens SBW25]	56	0.42
gi|229365022|emb|CAY53183.1|	putative sulfurtransferase [Pseudomonas fluorescens SBW25]	53	0.42
gi|229364202|emb|CAY51876.1|	pyruvate kinase [Pseudomonas fluorescens SBW25]	391	0.41
gi|229364294|emb|CAY52029.1|	inosine-5~-monophosphate dehydrogenase [Pseudomonas fluorescens SBW25]	166	0.41
gi|229362518|emb|CAY49425.1|	3-oxoacyl-[acyl-carrier-protein] synthase II [Pseudomonas fluorescens SBW25]	204	0.41
gi|229363944|emb|CAY51467.1|	electron transfer flavoprotein alpha-subunit [Pseudomonas fluorescens SBW25]	564	0.41
gi|229363740|emb|CAY51145.1|	aspartate aminotransferase [Pseudomonas fluorescens SBW25]	364	0.4
gi|229363900|emb|CAY51397.1|	putative carboxyvinyl-carboxyphosphonate phosphorylmutase [Pseudomonas fluorescens SBW25]	106	0.4
gi|229363669|emb|CAY51023.1|	pyoverdine synthetase A [Pseudomonas fluorescens SBW25]	6067	0.39
gi|229360677|emb|CAY47535.1|	putative surface exported protein [Pseudomonas fluorescens SBW25]	1138	0.39
gi|229360314|emb|CAY47171.1|	probable malate:quinone oxidoreductase [Pseudomonas fluorescens SBW25]	114	0.39
gi|229364996|emb|CAY53136.1|	dihydroorotase-like protein [Pseudomonas fluorescens SBW25]	735	0.39
gi|229363896|emb|CAY51390.1|	2-methylcitrate dehydratase [Pseudomonas fluorescens SBW25]	231	0.39
gi|229361114|emb|CAY47977.1|	probable protease (heat shock protein) [Pseudomonas fluorescens SBW25]	55	0.39
gi|229361847|emb|CAY48745.1|	putative TonB-receptor protein [Pseudomonas fluorescens SBW25]	674	0.38
gi|229363121|emb|CAY50140.1|	NADH dehydrogenase I chain C/D [Pseudomonas fluorescens SBW25]	224	0.38
gi|229359832|emb|CAY46682.1|	putative NADP-dependent malic enzyme [Pseudomonas fluorescens SBW25]	236	0.38
gi|229365065|emb|CAY53254.1|	D-3-phosphoglycerate dehydrogenase [Pseudomonas fluorescens SBW25]	194	0.38
gi|229362483|emb|CAY49389.1|	LysR-family transcriptional regulator [Pseudomonas fluorescens SBW25]	102	0.38
gi|229359779|emb|CAY46629.1|	glycogen phosphorylase [Pseudomonas fluorescens SBW25]	282	0.37
gi|229360628|emb|CAY47486.1|	lysyl-tRNA synthetase [Pseudomonas fluorescens SBW25]	126	0.37
gi|229360561|emb|CAY47418.1|	argininosuccinate synthase [Pseudomonas fluorescens SBW25]	113	0.37

gi|229360348|emb|CAY47205.1|	S-adenosyl-methyltransferase [Pseudomonas fluorescens SBW25]	90	0.37
gi|229364412|emb|CAY52207.1|	thioredoxin reductase [Pseudomonas fluorescens SBW25]	70	0.37
gi|229360286|emb|CAY47143.1|	superoxide dismutase [Mn] [Pseudomonas fluorescens SBW25]	599	0.37
gi|229364769|emb|CAY52766.1|	50S ribosomal protein L3 [Pseudomonas fluorescens SBW25]	245	0.37
gi|229363655|emb|CAY51001.1|	putative aspartyl aminopeptidase [Pseudomonas fluorescens SBW25]	134	0.36
gi|229360306|emb|CAY47163.1|	histidinol dehydrogenase [Pseudomonas fluorescens SBW25]	104	0.36
gi|229362021|emb|CAY48922.1|	putative 6-phosphogluconate dehydrogenase, decarboxylating [Pseudomonas fluorescens SBW25]	70	0.36
gi|229360493|emb|CAY47350.1|	putative TonB-dependent receptor [Pseudomonas fluorescens SBW25]	261	0.35
gi|229364572|emb|CAY52455.1|	NADP-specific glutamate dehydrogenase [Pseudomonas fluorescens SBW25]	296	0.35
gi|229363726|emb|CAY51125.1|	putative flagellar hook associated protein (FliD) [Pseudomonas fluorescens SBW25]	81	0.35
gi|229360663|emb|CAY47521.1|	putative lysine biosynthesis-related protein [Pseudomonas fluorescens SBW25]	152	0.35
gi|229362520|emb|CAY49427.1|	3-oxoacyl-[acyl-carrier-protein] synthase [Pseudomonas fluorescens SBW25]	141	0.35
gi|229364014|emb|CAY51571.1|	L-allo-threonine aldolase [Pseudomonas fluorescens SBW25]	151	0.35
gi|229364579|emb|CAY52465.1|	carbon starvation protein A [Pseudomonas fluorescens SBW25]	826	0.34
gi|229360331|emb|CAY47188.1|	putative medium-chain-fatty-acid--CoA ligase [Pseudomonas fluorescens SBW25]	595	0.34
gi|229360284|emb|CAY47141.1|	fumarate hydratase class II [Pseudomonas fluorescens SBW25]	539	0.34
gi|229364100|emb|CAY51711.1|	putative porin [Pseudomonas fluorescens SBW25]	416	0.34
gi|229360168|emb|CAY47025.1|	putative TonB-dependent exported protein [Pseudomonas fluorescens SBW25]	157	0.33
gi|229360235|emb|CAY47092.1|	putative outer membrane porin [Pseudomonas fluorescens SBW25]	474	0.33
gi|229360783|emb|CAY47641.1|	putative reductase [Pseudomonas fluorescens SBW25]	103	0.33
gi|229361515|emb|CAY48391.1|	probable succinyl-CoA:3-ketoacid-coenzyme A transferase subunit A [Pseudomonas fluorescens SBW25]	316	0.33
gi|229362266|emb|CAY49168.1|	putative TonB-dependent receptor YncD precursor [Pseudomonas fluorescens SBW25]	489	0.32
gi|229360271|emb|CAY47128.1|	glutamyl-tRNA(Gln) amidotransferase subunit A [Pseudomonas fluorescens SBW25]	162	0.32
gi|229363170|emb|CAY50215.1|	cysteinyl-tRNA synthetase [Pseudomonas fluorescens SBW25]	77	0.32
gi|229363488|emb|CAY50712.1|	putative 3-isopropylmalate dehydrogenase [Pseudomonas fluorescens SBW25]	145	0.32
gi|229361044|emb|CAY47906.1|	polysaccharide biosynthesis protein [Pseudomonas fluorescens SBW25]	92	0.32
gi|229361049|emb|CAY47912.1|	putative 3-oxoacyl-[acyl-carrier-protein] synthase III [Pseudomonas fluorescens SBW25]	78	0.32
gi|229363208|emb|CAY50280.1|	conserved hypothetical protein [Pseudomonas fluorescens SBW25]	250	0.32
gi|229360239|emb|CAY47096.1|	putative exported protein [Pseudomonas fluorescens SBW25]	234	0.32
gi|229362517|emb|CAY49424.1|	putative acyl CoA oxidase [Pseudomonas fluorescens SBW25]	676	0.31
gi|229364170|emb|CAY51826.1|	aspartyl-tRNA synthetase [Pseudomonas fluorescens SBW25]	392	0.31
gi|229359777|emb|CAY46627.1|	putative elongation facotr/GTP-binding protein TypA/BipA [Pseudomonas fluorescens SBW25]	569	0.31
gi|229359838|emb|CAY46690.1|	conserved hypothetical protein [Pseudomonas fluorescens SBW25]	139	0.31
gi|229359912|emb|CAY46766.1|	putative outer membrane protein [Pseudomonas fluorescens SBW25]	109	0.31
gi|229362548|emb|CAY49455.1|	putative secreted pectate lyase [Pseudomonas fluorescens SBW25]	426	0.31
gi|229364152|emb|CAY51794.1|	putative aminomethyltransferase (glycine cleavage system T protein) [Pseudomonas fluorescens SBW25]	141	0.31
gi|229360729|emb|CAY47587.1|	conserved hypothetical protein [Pseudomonas fluorescens SBW25]	67	0.31
gi|229362815|emb|CAY49725.1|	conserved hypothetical protein [Pseudomonas fluorescens SBW25]	331	0.3
gi|229359698|emb|CAY46546.1|	phosphoenolpyruvate carboxykinase [ATP] [Pseudomonas fluorescens SBW25]	303	0.3
gi|229361426|emb|CAY48301.1|	cysteine desulfurase [Pseudomonas fluorescens SBW25]	219	0.3

gi|229360943|emb|CAY47803.1|	3-ketoacyl-CoA thiolase [Pseudomonas fluorescens SBW25]	207	0.3
gi|229365106|emb|CAY53324.1|	putative aminotransferase [Pseudomonas fluorescens SBW25]	119	0.3
gi|229364304|emb|CAY52041.1|	conserved hypothetical protein [Pseudomonas fluorescens SBW25]	65	0.3
gi|229364030|emb|CAY51594.1|	acetyl-coenzyme A synthetase [Pseudomonas fluorescens SBW25]	124	0.29
gi|229363982|emb|CAY51521.1|	putative ribonuclease E [Pseudomonas fluorescens SBW25]	924	0.28
gi|229360222|emb|CAY47079.1|	putative ornithine decarboxylase [Pseudomonas fluorescens SBW25]	92	0.28
gi|229360636|emb|CAY47494.1|	putative lipoprotein [Pseudomonas fluorescens SBW25]	572	0.28
gi|229363883|emb|CAY51367.1|	putative membrane protein [Pseudomonas fluorescens SBW25]	464	0.28
gi|229359725|emb|CAY46573.1|	purative iron-transport related exported protein [Pseudomonas fluorescens SBW25]	386	0.27
gi|229364672|emb|CAY52606.1|	gamma-glutamyl phosphate reductase [Pseudomonas fluorescens SBW25]	246	0.27
gi|229360304|emb|CAY47161.1|	UDP-N-acetylglucosamine 1-carboxyvinyltransferase [Pseudomonas fluorescens SBW25]	152	0.27
gi|229364104|emb|CAY51718.1|	maltose-binding protein [Pseudomonas fluorescens SBW25]	95	0.27
gi|229361115|emb|CAY47978.1|	putative aminotransferase [Pseudomonas fluorescens SBW25]	73	0.27
gi|229364419|emb|CAY52219.1|	putative putative GTP-binding protein [Pseudomonas fluorescens SBW25]	68	0.27
gi|229364013|emb|CAY51570.1|	alanyl-tRNA synthetase [Pseudomonas fluorescens SBW25]	164	0.26
gi|229365226|emb|CAY53526.1|	guanosine-3~,5~-bis(diphosphate) 3~-pyrophosphohydrolase [Pseudomonas fluorescens SBW25]	118	0.26
gi|229359946|emb|CAY46800.1|	adenylosuccinate synthetase [Pseudomonas fluorescens SBW25]	81	0.26
gi|229362543|emb|CAY49450.1|	putative non-ribosomal peptide synthetase [Pseudomonas fluorescens SBW25]	886	0.25
gi|229365298|emb|CAY53646.1|	oxaloacetate decarboxylase alpha chain [Pseudomonas fluorescens SBW25]	84	0.25
gi|229361198|emb|CAY48062.1|	citrate synthase [Pseudomonas fluorescens SBW25]	251	0.25
gi|229364602|emb|CAY52500.1|	putative ABC transport system, outer membrane protein [Pseudomonas fluorescens SBW25]	97	0.25
gi|229363860|emb|CAY51327.1|	putative cell division protein [Pseudomonas fluorescens SBW25]	591	0.25
gi|229364606|emb|CAY52504.1|	putative TonB-dependent receptor protein [Pseudomonas fluorescens SBW25]	159	0.24
gi|229365055|emb|CAY53239.1|	phosphoenolpyruvate-protein phosphotransferase [Pseudomonas fluorescens SBW25]	272	0.24
gi|229364325|emb|CAY52076.1|	preprotein translocase, auxillary membrane component (General Secretory Pathway) [Pseudomonas fluorescens SBW25]	97	0.24
gi|229364247|emb|CAY51951.1|	proline tRNA synthetase [Pseudomonas fluorescens SBW25]	212	0.24
gi|229363802|emb|CAY51237.1|	ATP-dependent RNA helicase [Pseudomonas fluorescens SBW25]	63	0.24
gi|229365102|emb|CAY53317.1|	putative membrane protein [Pseudomonas fluorescens SBW25]	210	0.24
gi|229360362|emb|CAY47219.1|	UDP-3-O-[3-hydroxymyristoyl] N-acetylglucosamine deacetylase [Pseudomonas fluorescens SBW25]	210	0.24
gi|229360545|emb|CAY47402.1|	glutamate/aspartate ABC transport system, periplasmic binding protein precursor [Pseudomonas fluorescens SBW25]	192	0.24
gi|229361247|emb|CAY48111.1|	acyl-coenzyme A dehydrogenase [Pseudomonas fluorescens SBW25]	126	0.23
gi|229364834|emb|CAY52873.1|	RNA polymerase sigma factor (sigma-70) [Pseudomonas fluorescens SBW25]	288	0.23
gi|229360090|emb|CAY46944.1|	methylmalonate-semialdehyde dehydrogenase [Pseudomonas fluorescens SBW25]	223	0.23
gi|229361277|emb|CAY48141.1|	putative exported protein [Pseudomonas fluorescens SBW25]	161	0.23
gi|229363723|emb|CAY51119.1|	putative two-component system, response regulator nitrogen regulation protein NR(I) [Pseudomonas fluorescens SBW25]	325	0.22
gi|229364686|emb|CAY52632.1|	putative aldehyde dehydrigenase [Pseudomonas fluorescens SBW25]	158	0.22
gi|229361647|emb|CAY48527.1|	methylmalonate-semialdehyde dehydrogenase [Pseudomonas fluorescens SBW25]	84	0.22
gi|229362544|emb|CAY49451.1|	putative non-ribosomal peptide synthetase [Pseudomonas fluorescens SBW25]	823	0.21
gi|229364334|emb|CAY52089.1|	putative ATP-binding protein [Pseudomonas fluorescens SBW25]	137	0.21
gi|229362000|emb|CAY48901.1|	putative cobalamin biosynthesis-related protein [Pseudomonas fluorescens SBW25]	680	0.21

gi|229363125|emb|CAY50144.1|	NADH-quinone oxidoreductase chain H [Pseudomonas fluorescens SBW25]	269	0.21
gi|229365303|emb|CAY53652.1|	DNA helicase II [Pseudomonas fluorescens SBW25]	181	0.2
gi|229360237|emb|CAY47094.1|	dipeptide ABC transport system, substrate-binding protein [Pseudomonas fluorescens SBW25]	146	0.2
gi|229362324|emb|CAY49226.1|	putative 4-hydroxyphenylpyruvate dioxygenase [Pseudomonas fluorescens SBW25]	299	0.2
gi|229365160|emb|CAY53413.1|	transcriptional regulatory protein algp (alginate regulatory protein algr3) [Pseudomonas fluorescens SBW25]	246	0.2
gi|229362542|emb|CAY49449.1|	putative non-ribosomal peptide synthetase [Pseudomonas fluorescens SBW25]	816	0.19
gi|229362669|emb|CAY49579.1|	putative amidase [Pseudomonas fluorescens SBW25]	278	0.19
gi|229360447|emb|CAY47304.1|	iron (III) dicitrate transport protein precursor [Pseudomonas fluorescens SBW25]	82	0.19
gi|229361887|emb|CAY48787.1|	conserved hypothetical protein [Pseudomonas fluorescens SBW25]	389	0.19
gi|229360773|emb|CAY47631.1|	chaperone [Pseudomonas fluorescens SBW25]	61	0.19
gi|229360361|emb|CAY47218.1|	cell division protein [Pseudomonas fluorescens SBW25]	238	0.19
gi|229362214|emb|CAY49116.1|	conserved hypothetical protein [Pseudomonas fluorescens SBW25]	219	0.19
gi|229362541|emb|CAY49448.1|	putative non-ribosomal peptide synthetase [Pseudomonas fluorescens SBW25]	158	0.18
gi|229360465|emb|CAY47322.1|	GTP-binding protein [Pseudomonas fluorescens SBW25]	90	0.18
gi|229359480|emb|CAY46321.1|	tryptophan synthase beta chain [Pseudomonas fluorescens SBW25]	240	0.18
gi|229359945|emb|CAY46799.1|	putative ATP phosphoribosyltransferase regulatory subunit [Pseudomonas fluorescens SBW25]	225	0.18
gi|229364285|emb|CAY52013.1|	phosphoribosylformylglycinamidine synthase [Pseudomonas fluorescens SBW25]	358	0.17
gi|229362800|emb|CAY49710.1|	aconitate hydrase B [Pseudomonas fluorescens SBW25]	143	0.17
gi|229359586|emb|CAY46428.1|	conserved hypothetical protein [Pseudomonas fluorescens SBW25]	123	0.17
gi|229364502|emb|CAY52351.1|	protein chain initiation factor 2 [Pseudomonas fluorescens SBW25]	366	0.17
gi|229362012|emb|CAY48913.1|	putative aminopeptidase N [Pseudomonas fluorescens SBW25]	1392	0.16
gi|229360194|emb|CAY47051.1|	chaperone [Pseudomonas fluorescens SBW25]	284	0.16
gi|229364946|emb|CAY53056.1|	transketolase 1 [Pseudomonas fluorescens SBW25]	65	0.16
gi|229360068|emb|CAY46922.1|	putative short chain dehydrogenase/reductase [Pseudomonas fluorescens SBW25]	283	0.16
gi|229359492|emb|CAY46333.1|	oligopeptidase A [Pseudomonas fluorescens SBW25]	58	0.15
gi|229360775|emb|CAY47633.1|	acriflavine resistance protein B [Pseudomonas fluorescens SBW25]	1003	0.14
gi|229363106|emb|CAY50113.1|	ATP-dependent Clp protease ATP-binding subunit ClpA [Pseudomonas fluorescens SBW25]	71	0.14
gi|229363501|emb|CAY50735.1|	putative glutamyl-tRNA synthetase [Pseudomonas fluorescens SBW25]	328	0.14
gi|229363226|emb|CAY50309.1|	DNA-binding ATP-dependent protease La; heat shock K-protein [Pseudomonas fluorescens SBW25]	128	0.13
gi|229363440|emb|CAY50635.1|	phenylalanyl-tRNA synthetase beta chain [Pseudomonas fluorescens SBW25]	87	0.13
gi|229360492|emb|CAY47349.1|	quinoprotein glucose dehydrogenase [Pseudomonas fluorescens SBW25]	82	0.13
gi|229362374|emb|CAY49276.1|	putative methyl-accepting chemotaxis protein [Pseudomonas fluorescens SBW25]	263	0.13
gi|229360748|emb|CAY47606.1|	RNA polymerase associated protein [Pseudomonas fluorescens SBW25]	148	0.11
gi|229364426|emb|CAY52231.1|	methyl-accepting chemotaxis protein II [Pseudomonas fluorescens SBW25]	259	0.11
gi|229364514|emb|CAY52369.1|	carbamoyl-phosphate synthase large chain [Pseudomonas fluorescens SBW25]	77	0.1
gi|229359884|emb|CAY46737.1|	putative glutamate-ammonia-ligase adenylyltransferase [Pseudomonas fluorescens SBW25]	72	0.1
gi|229362368|emb|CAY49270.1|	putative formyl transferase [Pseudomonas fluorescens SBW25]	612	0.1
gi|229365244|emb|CAY53554.1|	putative membrane protein [Pseudomonas fluorescens SBW25]	57	0.09
gi|229359874|emb|CAY46727.1|	bifunctional PutA protein [Pseudomonas fluorescens SBW25]	69	0.08
gi|229361030|emb|CAY47892.1|	DNA gyrase subunit A [Pseudomonas fluorescens SBW25]	260	0.08

gi|229359839|emb|CAY46691.1|	glutamate synthase [NADPH] large chain precursor [Pseudomonas fluorescens SBW25]	75	0.07
gi|229362510|emb|CAY49417.1|	putative adhesin [Pseudomonas fluorescens SBW25]	177	0.04
gi|229362566|emb|CAY49473.1|	putative adhesin [Pseudomonas fluorescens SBW25]	152	0.03

Biofilm MV sample

Note: Human contaminants removed.

ID	Protein	prot_score	emPAI
gi|229363728|emb|CAY51128.1|	flagellin [Pseudomonas fluorescens SBW25]	28622	5.36
gi|229361881|emb|CAY48781.1|	ferripyoverdine receptor precursor [Pseudomonas fluorescens SBW25]	28858	4.75
gi|229364161|emb|CAY51810.1|	TolB colicin import protein [Pseudomonas fluorescens SBW25]	5548	4.29
gi|229364712|emb|CAY52676.1|	6,7-dimethyl-8-ribityllumazine synthase [Pseudomonas fluorescens SBW25]	685	2.57
gi|229360670|emb|CAY47528.1|	elongation factor [Pseudomonas fluorescens SBW25]	663	2.24
gi|229364221|emb|CAY51907.1|	putative exported heme receptor protein [Pseudomonas fluorescens SBW25]	15939	2.2
gi|229364725|emb|CAY52696.1|	putative ABC transport system, outer membrane exported protein [Pseudomonas fluorescens SBW25]	2128	2.16
gi|229360345|emb|CAY47202.1|	putative lipoprotein [Pseudomonas fluorescens SBW25]	5246	1.87
gi|229360318|emb|CAY47175.1|	putative lipoprotein [Pseudomonas fluorescens SBW25]	4312	1.8
gi|229362537|emb|CAY49444.1|	putative TonB-dependent outer membrane receptor [Pseudomonas fluorescens SBW25]	3229	1.73
gi|229364468|emb|CAY52297.1|	ketol-acid reductoisomerase [Pseudomonas fluorescens SBW25]	1248	1.69
gi|229359762|emb|CAY46612.1|	carboxy-terminal processing protease precursor [Pseudomonas fluorescens SBW25]	1905	1.54
gi|229360012|emb|CAY46866.1|	putative outer membrane protein [Pseudomonas fluorescens SBW25]	3423	1.51
gi|229360407|emb|CAY47264.1|	putative amino acid ABC transport system, substrate-binding protein [Pseudomonas fluorescens SBW25]	2088	1.41
gi|229360727|emb|CAY47585.1|	putative membrane protein [Pseudomonas fluorescens SBW25]	684	1.4
gi|229360636|emb|CAY47494.1|	putative lipoprotein [Pseudomonas fluorescens SBW25]	3075	1.39
gi|229364178|emb|CAY51840.1|	putative family S43 non-peptidase homologue [Pseudomonas fluorescens SBW25]	2487	1.34
gi|229363881|emb|CAY51363.1|	putative outer membrane porin A protein [Pseudomonas fluorescens SBW25]	9019	1.29
gi|229364240|emb|CAY51939.1|	chaperone Hsp60, peptide-dependent ATPase, heat shock protein [Pseudomonas fluorescens SBW25]	2691	1.28
gi|229364385|emb|CAY52165.1|	putative aminotransferase [Pseudomonas fluorescens SBW25]	1289	1.27
gi|229361136|emb|CAY47999.1|	putative lipoprotein [Pseudomonas fluorescens SBW25]	561	1.27
gi|229365095|emb|CAY53304.1|	putative outer membrane protein [Pseudomonas fluorescens SBW25]	4165	1.24
gi|229364160|emb|CAY51808.1|	putative OmpA family lipoprotein [Pseudomonas fluorescens SBW25]	1561	1.21
gi|229364173|emb|CAY51832.1|	putative ferritin DPS-family DNA binding protein [Pseudomonas fluorescens SBW25]	557	1.2
gi|229359915|emb|CAY46769.1|	putative lipoprotein [Pseudomonas fluorescens SBW25]	655	1.17
gi|229359726|emb|CAY46574.1|	outer membrane protein, oprE3 precursor [Pseudomonas fluorescens SBW25]	3162	1.15
gi|229364115|emb|CAY51736.1|	putative exported protein [Pseudomonas fluorescens SBW25]	2894	1.14
gi|229364029|emb|CAY51593.1|	putative histidine-binding periplasmic protein precursor [Pseudomonas fluorescens SBW25]	330	1.12
gi|229359677|emb|CAY46525.1|	putative ABC transport system, exported protein [Pseudomonas fluorescens SBW25]	930	1.11
gi|229363756|emb|CAY51170.1|	putative fatty acid transport system, membrane protein [Pseudomonas fluorescens SBW25]	794	1.02
gi|229360239|emb|CAY47096.1|	putative exported protein [Pseudomonas fluorescens SBW25]	334	1
gi|229364518|emb|CAY52373.1|	chaperone protein DnkA (HSP70) [Pseudomonas fluorescens SBW25]	3014	0.99
gi|229360462|emb|CAY47319.1|	cytosol aminopeptidase [Pseudomonas fluorescens SBW25]	1519	0.99
gi|229364964|emb|CAY53084.1|	putative exported protein [Pseudomonas fluorescens SBW25]	397	0.98
gi|229360548|emb|CAY47405.1|	glycerol kinase [Pseudomonas fluorescens SBW25]	1477	0.92
gi|229365349|emb|CAY53727.1|	ATP synthase alpha chain [Pseudomonas fluorescens SBW25]	971	0.92

gi|229363458|emb|CAY50667.1|	putative L-asparaginase II precursor [Pseudomonas fluorescens SBW25]	891	0.91
gi|229363134|emb|CAY50159.1|	conserved hypothetical protein [Pseudomonas fluorescens SBW25]	599	0.91
gi|229362693|emb|CAY49603.1|	putative ferric alcaligin siderophore receptor [Pseudomonas fluorescens SBW25]	2877	0.9
gi|229359800|emb|CAY46650.1|	glucans biosynthesis protein G precursor [Pseudomonas fluorescens SBW25]	1863	0.88
gi|229362018|emb|CAY48919.1|	putative ferric alcaligin siderophore receptor [Pseudomonas fluorescens SBW25]	1644	0.85
gi|229361847|emb|CAY48745.1|	putative TonB-receptor protein [Pseudomonas fluorescens SBW25]	1618	0.83
gi|229361207|emb|CAY48071.1|	succinyl-CoA synthetase alpha chain [Pseudomonas fluorescens SBW25]	436	0.81
gi|229364778|emb|CAY52781.1|	50S ribosomal protein L10 [Pseudomonas fluorescens SBW25]	55	0.81
gi|229364779|emb|CAY52783.1|	50S ribosomal protein L1 [Pseudomonas fluorescens SBW25]	422	0.8
gi|229365347|emb|CAY53725.1|	ATP synthase beta chain [Pseudomonas fluorescens SBW25]	1049	0.79
gi|229364770|emb|CAY52768.1|	30S ribosomal protein S10 [Pseudomonas fluorescens SBW25]	70	0.79
gi|229364100|emb|CAY51711.1|	putative porin [Pseudomonas fluorescens SBW25]	790	0.78
gi|229363660|emb|CAY51010.1|	diaminobutyrate--2-oxoglutarate aminotransferase [Pseudomonas fluorescens SBW25]	767	0.78
gi|229360493|emb|CAY47350.1|	putative TonB-dependent receptor [Pseudomonas fluorescens SBW25]	1202	0.74
gi|229360153|emb|CAY47010.1|	putative exported protein [Pseudomonas fluorescens SBW25]	149	0.74
gi|229360678|emb|CAY47536.1|	putative outer membrane protein [Pseudomonas fluorescens SBW25]	117	0.74
gi|229364606|emb|CAY52504.1|	putative TonB-dependent receptor protein [Pseudomonas fluorescens SBW25]	886	0.71
gi|229360545|emb|CAY47402.1|	glutamate/aspartate ABC transport system, periplasmic binding protein precursor [Pseudomonas fluorescens SBW25]	1311	0.71
gi|229365228|emb|CAY53529.1|	putative lipoprotein [Pseudomonas fluorescens SBW25]	342	0.71
gi|229361206|emb|CAY48070.1|	succinyl-CoA synthetase beta chain [Pseudomonas fluorescens SBW25]	1392	0.68
gi|229362266|emb|CAY49168.1|	putative TonB-dependent receptor YncD precursor [Pseudomonas fluorescens SBW25]	1251	0.67
gi|229359512|emb|CAY46353.1|	putative D-methionine ABC transport system, substrate-binding protein [Pseudomonas fluorescens SBW25]	78	0.67
gi|229360333|emb|CAY47190.1|	putative exported protein [Pseudomonas fluorescens SBW25]	123	0.65
gi|229362520|emb|CAY49427.1|	3-oxoacyl-[acyl-carrier-protein] synthase [Pseudomonas fluorescens SBW25]	123	0.65
gi|229360663|emb|CAY47521.1|	putative lysine biosynthesis-related protein [Pseudomonas fluorescens SBW25]	113	0.64
gi|229363451|emb|CAY50654.1|	putative inosine-uridine preferring nucleoside hydrolase [Pseudomonas fluorescens SBW25]	559	0.63
gi|229360143|emb|CAY47000.1|	putative ribosomal protein L25p family [Pseudomonas fluorescens SBW25]	81	0.63
gi|229363996|emb|CAY51545.1|	putative flagellar hook protein [Pseudomonas fluorescens SBW25]	375	0.61
gi|229364145|emb|CAY51782.1|	ornithine carbamoyltransferase, catabolic [Pseudomonas fluorescens SBW25]	617	0.61
gi|229360742|emb|CAY47600.1|	branched amino acid ABC transport system, substrate-binding protein [Pseudomonas fluorescens SBW25]	614	0.57
gi|229360870|emb|CAY47729.1|	putative lipoprotein [Pseudomonas fluorescens SBW25]	742	0.56
gi|229360807|emb|CAY47665.1|	probable alcohol dehydrogenase [Pseudomonas fluorescens SBW25]	132	0.56
gi|229359963|emb|CAY46817.1|	azurin precursor [Pseudomonas fluorescens SBW25]	142	0.55
gi|229363585|emb|CAY50877.1|	putative putative heat shock protein A [Pseudomonas fluorescens SBW25]	587	0.52
gi|229361277|emb|CAY48141.1|	putative exported protein [Pseudomonas fluorescens SBW25]	192	0.51
gi|229363945|emb|CAY51470.1|	electron transfer flavoprotein beta-subunit [Pseudomonas fluorescens SBW25]	246	0.5
gi|229364773|emb|CAY52773.1|	30S ribosomal protein S7 [Pseudomonas fluorescens SBW25]	62	0.49
gi|229365027|emb|CAY53190.1|	phosphopantetheine adenylyltransferase [Pseudomonas fluorescens SBW25]	68	0.48
gi|229364498|emb|CAY52345.1|	polynucleotide phosphorylase/polyadenylase [Pseudomonas fluorescens SBW25]	264	0.47
gi|229363775|emb|CAY51194.1|	conserved hypothetical protein [Pseudomonas fluorescens SBW25]	587	0.47

gi|229360168|emb|CAY47025.1|	putative TonB-dependent exported protein [Pseudomonas fluorescens SBW25]	403	0.46
gi|229359725|emb|CAY46573.1|	purative iron-transport related exported protein [Pseudomonas fluorescens SBW25]	346	0.46
gi|229364941|emb|CAY53048.1|	fructose-bisphosphate aldolase [Pseudomonas fluorescens SBW25]	89	0.45
gi|229360326|emb|CAY47183.1|	putative exported peptidase [Pseudomonas fluorescens SBW25]	273	0.44
gi|229364754|emb|CAY52740.1|	50S ribosomal protein L6 [Pseudomonas fluorescens SBW25]	73	0.44
gi|229360235|emb|CAY47092.1|	putative outer membrane porin [Pseudomonas fluorescens SBW25]	890	0.43
gi|229360863|emb|CAY47722.1|	putative exported heat-shock adaptation serine protease [Pseudomonas fluorescens SBW25]	974	0.43
gi|229361513|emb|CAY48389.1|	putative acetyl-CoA transferase [Pseudomonas fluorescens SBW25]	473	0.43
gi|229359803|emb|CAY46653.1|	putative ABC transport system, exported protein [Pseudomonas fluorescens SBW25]	174	0.43
gi|229361985|emb|CAY48885.1|	conserved hypothetical protein [Pseudomonas fluorescens SBW25]	121	0.43
gi|229362669|emb|CAY49579.1|	putative amidase [Pseudomonas fluorescens SBW25]	992	0.42
gi|229364304|emb|CAY52041.1|	conserved hypothetical protein [Pseudomonas fluorescens SBW25]	224	0.42
gi|229364817|emb|CAY52842.1|	putative exported organic solvent tolerance protein [Pseudomonas fluorescens SBW25]	203	0.41
gi|229360534|emb|CAY47391.1|	putative aminotransferase [Pseudomonas fluorescens SBW25]	663	0.41
gi|229362312|emb|CAY49214.1|	UTP--glucose-1-phosphate uridylyltransferase [Pseudomonas fluorescens SBW25]	220	0.41
gi|229364757|emb|CAY52745.1|	50S ribosomal protein L5 [Pseudomonas fluorescens SBW25]	123	0.41
gi|229360341|emb|CAY47198.1|	putative phospholipid-binding lipoprotein [Pseudomonas fluorescens SBW25]	64	0.41
gi|229362316|emb|CAY49218.1|	alkyl hydroperoxide reductase protein [Pseudomonas fluorescens SBW25]	41	0.41
gi|229360147|emb|CAY47004.1|	conserved hypothetical protein [Pseudomonas fluorescens SBW25]	399	0.4
gi|229361408|emb|CAY48283.1|	putative ABC transport system, substrate-binding protein [Pseudomonas fluorescens SBW25]	68	0.4
gi|229364519|emb|CAY52374.1|	protein GrpE (HSP-70 cofactor) [Pseudomonas fluorescens SBW25]	239	0.4
gi|229363896|emb|CAY51390.1|	2-methylcitrate dehydratase [Pseudomonas fluorescens SBW25]	276	0.39
gi|229363478|emb|CAY50697.1|	O-succinylhomoserine sulfhydrylase [Pseudomonas fluorescens SBW25]	168	0.39
gi|229361099|emb|CAY47962.1|	putative elongation factor [Pseudomonas fluorescens SBW25]	110	0.39
gi|229365237|emb|CAY53542.1|	conserved hypothetical protein [Pseudomonas fluorescens SBW25]	287	0.38
gi|229360557|emb|CAY47414.1|	putative alkyl hydroperoxide reductase [Pseudomonas fluorescens SBW25]	320	0.38
gi|229362957|emb|CAY49887.1|	putative peptidase [Pseudomonas fluorescens SBW25]	133	0.37
gi|229362643|emb|CAY49551.1|	putative rhizopine biosynthesis/dihydropicolinate synthase [Pseudomonas fluorescens SBW25]	76	0.37
gi|229364769|emb|CAY52766.1|	50S ribosomal protein L3 [Pseudomonas fluorescens SBW25]	89	0.37
gi|229363655|emb|CAY51001.1|	putative aspartyl aminopeptidase [Pseudomonas fluorescens SBW25]	132	0.36
gi|229360146|emb|CAY47003.1|	putative outer-membrane lipoprotein [Pseudomonas fluorescens SBW25]	336	0.36
gi|229360305|emb|CAY47162.1|	ATP phosphoribosyltransferase [Pseudomonas fluorescens SBW25]	39	0.36
gi|229361213|emb|CAY48077.1|	chaperone (heat shock protein) [Pseudomonas fluorescens SBW25]	124	0.35
gi|229364248|emb|CAY51953.1|	putative lipoprotein [Pseudomonas fluorescens SBW25]	307	0.35
gi|229364492|emb|CAY52336.1|	glucose-6-phosphate isomerase [Pseudomonas fluorescens SBW25]	225	0.34
gi|229360284|emb|CAY47141.1|	fumarate hydratase class II [Pseudomonas fluorescens SBW25]	581	0.34
gi|229364818|emb|CAY52844.1|	putative chaperone [Pseudomonas fluorescens SBW25]	347	0.34
gi|229364744|emb|CAY52723.1|	DNA-directed RNA polymerase alpha chain [Pseudomonas fluorescens SBW25]	195	0.34
gi|229364790|emb|CAY52800.1|	N-acetyl-gamma-glutamyl-phosphate reductase [Pseudomonas fluorescens SBW25]	101	0.34
gi|229359606|emb|CAY46448.1|	putative lipoprotein [Pseudomonas fluorescens SBW25]	53	0.34

gi|229360942|emb|CAY47802.1|	fatty oxidation complex alpha subunit [Pseudomonas fluorescens SBW25]	126	0.33
gi|229361205|emb|CAY48069.1|	dihydrolipoyl dehydrogenase [Pseudomonas fluorescens SBW25]	173	0.33
gi|229364959|emb|CAY53075.1|	adenosylhomocysteinase [Pseudomonas fluorescens SBW25]	110	0.33
gi|229360191|emb|CAY47048.1|	putative lipoprotein [Pseudomonas fluorescens SBW25]	478	0.32
gi|229363973|emb|CAY51508.1|	3-oxoacyl-[acyl-carrier protein] reductase [Pseudomonas fluorescens SBW25]	54	0.32
gi|229365019|emb|CAY53178.1|	conserved hypothetical protein [Pseudomonas fluorescens SBW25]	293	0.31
gi|229359912|emb|CAY46766.1|	putative outer membrane protein [Pseudomonas fluorescens SBW25]	136	0.31
gi|229363297|emb|CAY50405.1|	putative exported protein [Pseudomonas fluorescens SBW25]	113	0.31
gi|229365081|emb|CAY53280.1|	putrescine ABC transport system, substrate-binding periplasmic protein precursor [Pseudomonas fluorescens SBW25]	374	0.31
gi|229363220|emb|CAY50301.1|	putative lipoprotein [Pseudomonas fluorescens SBW25]	434	0.3
gi|229359974|emb|CAY46828.1|	putative amino acid oxidase [Pseudomonas fluorescens SBW25]	401	0.3
gi|229360709|emb|CAY47567.1|	putative amino acid transport system, substrate-binding protein [Pseudomonas fluorescens SBW25]	64	0.3
gi|229359882|emb|CAY46735.1|	dihydrolipoyllysine-residue acetyltransferase component of pyruvate dehydrogenase complex [Pseudomonas fluorescens SBW	382	0.29
gi|229362357|emb|CAY49259.1|	putative phenylacetic acid degradation enoyl-CoA hydratase [Pseudomonas fluorescens SBW25]	215	0.29
gi|229359741|emb|CAY46590.1|	putative amino acid ABC transport system, membrane protein [Pseudomonas fluorescens SBW25]	81	0.29
gi|229364150|emb|CAY51790.1|	glycine dehydrogenase [decarboxylating] [Pseudomonas fluorescens SBW25]	550	0.28
gi|229360677|emb|CAY47535.1|	putative surface exported protein [Pseudomonas fluorescens SBW25]	573	0.28
gi|229364771|emb|CAY52770.1|	elongation factor TU [Pseudomonas fluorescens SBW25]	533	0.28
gi|229363503|emb|CAY50737.1|	aromatic-amino-acid aminotransferase [Pseudomonas fluorescens SBW25]	177	0.28
gi|229364159|emb|CAY51806.1|	putative exported protein [Pseudomonas fluorescens SBW25]	373	0.28
gi|229359623|emb|CAY46465.1|	4-aminobutyrate aminotransferase [Pseudomonas fluorescens SBW25]	202	0.27
gi|229364672|emb|CAY52606.1|	gamma-glutamyl phosphate reductase [Pseudomonas fluorescens SBW25]	98	0.27
gi|229364012|emb|CAY51568.1|	aspartokinase [Pseudomonas fluorescens SBW25]	86	0.27
gi|229364766|emb|CAY52761.1|	50S ribosomal protein L2 [Pseudomonas fluorescens SBW25]	160	0.27
gi|229361927|emb|CAY48827.1|	putative TonB-dependent receptor [Pseudomonas fluorescens SBW25]	309	0.26
gi|229364772|emb|CAY52772.1|	elongation factor G [Pseudomonas fluorescens SBW25]	249	0.26
gi|229361242|emb|CAY48106.1|	putative dipeptidase [Pseudomonas fluorescens SBW25]	72	0.26
gi|229359883|emb|CAY46736.1|	pyruvate dehydrogenase E1 component [Pseudomonas fluorescens SBW25]	571	0.25
gi|229365298|emb|CAY53646.1|	oxaloacetate decarboxylase alpha chain [Pseudomonas fluorescens SBW25]	87	0.25
gi|229364659|emb|CAY52587.1|	putative lipoprotein [Pseudomonas fluorescens SBW25]	108	0.25
gi|229363229|emb|CAY50312.1|	trigger factor [Pseudomonas fluorescens SBW25]	104	0.25
gi|229365218|emb|CAY53512.1|	acetylglutamate kinase [Pseudomonas fluorescens SBW25]	110	0.25
gi|229360447|emb|CAY47304.1|	iron (III) dicitrate transport protein precursor [Pseudomonas fluorescens SBW25]	298	0.24
gi|229365018|emb|CAY53176.1|	putative peptidase [Pseudomonas fluorescens SBW25]	112	0.24
gi|229365217|emb|CAY53511.1|	putative phosphomannomutase/phosphoglucomutase [Pseudomonas fluorescens SBW25]	67	0.24
gi|229363114|emb|CAY50127.1|	adenylosuccinate lyase [Pseudomonas fluorescens SBW25]	59	0.24
gi|229361917|emb|CAY48817.1|	putative rhizopine-binding ABC transporter protein [Pseudomonas fluorescens SBW25]	127	0.24
gi|229363457|emb|CAY50665.1|	putative ribose transport system, substrate-binding protein [Pseudomonas fluorescens SBW25]	101	0.24
gi|229361086|emb|CAY47949.1|	conserved hypothetical exported protein [Pseudomonas fluorescens SBW25]	85	0.24
gi|229363482|emb|CAY50703.1|	acetyl CoA carboxylase, carboxyltranferase subunit beta [Pseudomonas fluorescens SBW25]	64	0.24

gi|229363888|emb|CAY51376.1|	putative phosphoenolpyruvate synthase [Pseudomonas fluorescens SBW25]	294	0.23
gi|229359775|emb|CAY46625.1|	glutamine synthetase [Pseudomonas fluorescens SBW25]	404	0.23
gi|229365169|emb|CAY53427.1|	argininosuccinate lyase [Pseudomonas fluorescens SBW25]	85	0.23
gi|229364422|emb|CAY52224.1|	octaprenyl-diphosphate synthase [Pseudomonas fluorescens SBW25]	42	0.23
gi|229362368|emb|CAY49270.1|	putative formyl transferase [Pseudomonas fluorescens SBW25]	220	0.22
gi|229364218|emb|CAY51902.1|	glyceraldehyde 3-phosphate dehydrogenase 1 [Pseudomonas fluorescens SBW25]	160	0.22
gi|229364995|emb|CAY53133.1|	aspartate carbamoyltransferase [Pseudomonas fluorescens SBW25]	62	0.22
gi|229361717|emb|CAY48598.1|	putative TonB-dependent siderophore receptor [Pseudomonas fluorescens SBW25]	80	0.2
gi|229364293|emb|CAY52028.1|	GMP synthase (glutamine-hydrolyzing) [Pseudomonas fluorescens SBW25]	81	0.2
gi|229360610|emb|CAY47467.1|	glucans biosynthesis protein D precursor [Pseudomonas fluorescens SBW25]	68	0.2
gi|229359969|emb|CAY46823.1|	putative branched amino acid ABC transport system, substrate-binding protein [Pseudomonas fluorescens SBW25]	232	0.2
gi|229363488|emb|CAY50712.1|	putative 3-isopropylmalate dehydrogenase [Pseudomonas fluorescens SBW25]	79	0.2
gi|229365160|emb|CAY53413.1|	transcriptional regulatory protein algp (alginate regulatory protein algr3) [Pseudomonas fluorescens SBW25]	72	0.2
gi|229364152|emb|CAY51794.1|	putative aminomethyltransferase (glycine cleavage system T protein) [Pseudomonas fluorescens SBW25]	54	0.2
gi|229360794|emb|CAY47652.1|	periplasmic beta-glucosidase precursor [Pseudomonas fluorescens SBW25]	122	0.19
gi|229362548|emb|CAY49455.1|	putative secreted pectate lyase [Pseudomonas fluorescens SBW25]	544	0.19
gi|229360943|emb|CAY47803.1|	3-ketoacyl-CoA thiolase [Pseudomonas fluorescens SBW25]	162	0.19
gi|229364300|emb|CAY52035.1|	2-isopropylmalate synthase [Pseudomonas fluorescens SBW25]	126	0.19
gi|229363487|emb|CAY50710.1|	aspartate-semialdehyde dehydrogenase [Pseudomonas fluorescens SBW25]	93	0.19
gi|229362518|emb|CAY49425.1|	3-oxoacyl-[acyl-carrier-protein] synthase II [Pseudomonas fluorescens SBW25]	77	0.19
gi|229362358|emb|CAY49260.1|	acyl-CoA dehydrogenase [Pseudomonas fluorescens SBW25]	64	0.19
gi|229359476|emb|CAY46317.1|	galactonate dehydratase [Pseudomonas fluorescens SBW25]	64	0.19
gi|229364515|emb|CAY52370.1|	carbamoyl-phosphate synthase small chain [Pseudomonas fluorescens SBW25]	63	0.19
gi|229364664|emb|CAY52594.1|	putative cell division-related protein [Pseudomonas fluorescens SBW25]	53	0.19
gi|229364775|emb|CAY52776.1|	DNA-directed RNA polymerase beta~ chain [Pseudomonas fluorescens SBW25]	310	0.18
gi|229360332|emb|CAY47189.1|	conserved hypothetical protein [Pseudomonas fluorescens SBW25]	250	0.18
gi|229364996|emb|CAY53136.1|	dihydroorotase-like protein [Pseudomonas fluorescens SBW25]	90	0.18
gi|229363188|emb|CAY50246.1|	putative acyl-CoA dehydrogenase [Pseudomonas fluorescens SBW25]	65	0.18
gi|229363731|emb|CAY51133.1|	putative flagellar hook-associated protein [Pseudomonas fluorescens SBW25]	79	0.17
gi|229359832|emb|CAY46682.1|	putative NADP-dependent malic enzyme [Pseudomonas fluorescens SBW25]	228	0.17
gi|229363109|emb|CAY50118.1|	isocitrate dehydrogenase [NADP] [Pseudomonas fluorescens SBW25]	144	0.17
gi|229360689|emb|CAY47547.1|	enolase [Pseudomonas fluorescens SBW25]	81	0.17
gi|229359946|emb|CAY46800.1|	adenylosuccinate synthetase [Pseudomonas fluorescens SBW25]	63	0.17
gi|229362026|emb|CAY48927.1|	putative exported protein [Pseudomonas fluorescens SBW25]	60	0.17
gi|229359825|emb|CAY46675.1|	ATP-dependent hsl protease ATP-binding subunit [Pseudomonas fluorescens SBW25]	46	0.16
gi|229359622|emb|CAY46464.1|	succinate-semialdehyde dehydrogenase [NADP+] [Pseudomonas fluorescens SBW25]	101	0.15
gi|229362514|emb|CAY49421.1|	putative FAD-binding oxidase [Pseudomonas fluorescens SBW25]	96	0.15
gi|229365209|emb|CAY53495.1|	putative aldehyde dehydrogenase [Pseudomonas fluorescens SBW25]	71	0.15
gi|229364202|emb|CAY51876.1|	pyruvate kinase [Pseudomonas fluorescens SBW25]	51	0.15
gi|229365064|emb|CAY53252.1|	putative oxidase [Pseudomonas fluorescens SBW25]	48	0.15

gi|229361024|emb|CAY47886.1|	putative peptide ABC transport system, substrate-binding protein [Pseudomonas fluorescens SBW25]	51	0.14
gi|229362022|emb|CAY48923.1|	glucose-6-phosphate 1-dehydrogenase [Pseudomonas fluorescens SBW25]	33	0.14
gi|229364870|emb|CAY52932.1|	putative TonB-dependent membrane protein [Pseudomonas fluorescens SBW25]	265	0.13
gi|229364502|emb|CAY52351.1|	protein chain initiation factor 2 [Pseudomonas fluorescens SBW25]	92	0.13
gi|229359586|emb|CAY46428.1|	conserved hypothetical protein [Pseudomonas fluorescens SBW25]	70	0.13
gi|229363226|emb|CAY50309.1|	DNA-binding ATP-dependent protease La; heat shock K-protein [Pseudomonas fluorescens SBW25]	59	0.13
gi|229360237|emb|CAY47094.1|	dipeptide ABC transport system, substrate-binding protein [Pseudomonas fluorescens SBW25]	85	0.13
gi|229365213|emb|CAY53502.1|	putative transport-related, exported protein [Pseudomonas fluorescens SBW25]	56	0.13
gi|229361201|emb|CAY48065.1|	succinate dehydrogenase flavoprotein subunit [Pseudomonas fluorescens SBW25]	143	0.12
gi|229360121|emb|CAY46975.1|	putative lipoprotein [Pseudomonas fluorescens SBW25]	113	0.12
gi|229361036|emb|CAY47898.1|	30S ribosomal protein S1 [Pseudomonas fluorescens SBW25]	57	0.12
gi|229361427|emb|CAY48302.1|	putative peptidase [Pseudomonas fluorescens SBW25]	44	0.12
gi|229359828|emb|CAY46678.1|	arginyl-tRNA synthetase [Pseudomonas fluorescens SBW25]	40	0.12
gi|229365033|emb|CAY53201.1|	dihydroxy-acid dehydratase [Pseudomonas fluorescens SBW25]	35	0.12
gi|229359777|emb|CAY46627.1|	putative elongation facotr/GTP-binding protein TypA/BipA [Pseudomonas fluorescens SBW25]	63	0.11
gi|229363982|emb|CAY51521.1|	putative ribonuclease E [Pseudomonas fluorescens SBW25]	85	0.1
gi|229359492|emb|CAY46333.1|	oligopeptidase A [Pseudomonas fluorescens SBW25]	61	0.1
gi|229359576|emb|CAY46418.1|	putative exported protein [Pseudomonas fluorescens SBW25]	44	0.1
gi|229363110|emb|CAY50120.1|	isocitrate dehydrogenase [NADP] [Pseudomonas fluorescens SBW25]	81	0.09
gi|229360429|emb|CAY47286.1|	putative ferric siderophore receptor [Pseudomonas fluorescens SBW25]	59	0.09
gi|229361030|emb|CAY47892.1|	DNA gyrase subunit A [Pseudomonas fluorescens SBW25]	55	0.08
gi|229362800|emb|CAY49710.1|	aconitate hydrase B [Pseudomonas fluorescens SBW25]	41	0.08
gi|229364776|emb|CAY52777.1|	DNA-directed RNA polymerase beta chain [Pseudomonas fluorescens SBW25]	82	0.05
gi|229364285|emb|CAY52013.1|	phosphoribosylformylglycinamidine synthase [Pseudomonas fluorescens SBW25]	74	0.05
gi|229362815|emb|CAY49725.1|	conserved hypothetical protein [Pseudomonas fluorescens SBW25]	305	0.04
gi|229359591|emb|CAY46433.1|	putative adhesin [Pseudomonas fluorescens SBW25]	34	0.03

Plankton MV sample

Note: Human contaminants removed.

ID	Protein	prot_score	emPAI
gi|229364773|emb|CAY52773.1|	30S ribosomal protein S7 [Pseudomonas fluorescens SBW25]	1211	2.28
gi|229364161|emb|CAY51810.1|	TolB colicin import protein [Pseudomonas fluorescens SBW25]	2081	1.89
gi|229364145|emb|CAY51782.1|	ornithine carbamoyltransferase, catabolic [Pseudomonas fluorescens SBW25]	969	1.83
gi|229364240|emb|CAY51939.1|	chaperone Hsp60, peptide-dependent ATPase, heat shock protein [Pseudomonas fluorescens SBW25]	3493	1.76
gi|229361881|emb|CAY48781.1|	ferripyoverdine receptor precursor [Pseudomonas fluorescens SBW25]	3600	1.55
gi|229364221|emb|CAY51907.1|	putative exported heme receptor protein [Pseudomonas fluorescens SBW25]	5245	1.54
gi|229362537|emb|CAY49444.1|	putative TonB-dependent outer membrane receptor [Pseudomonas fluorescens SBW25]	1806	1.36
gi|229363728|emb|CAY51128.1|	flagellin [Pseudomonas fluorescens SBW25]	2980	1.26
gi|229359762|emb|CAY46612.1|	carboxy-terminal processing protease precursor [Pseudomonas fluorescens SBW25]	1205	1.17
gi|229365095|emb|CAY53304.1|	putative outer membrane protein [Pseudomonas fluorescens SBW25]	2262	1.08
gi|229360012|emb|CAY46866.1|	putative outer membrane protein [Pseudomonas fluorescens SBW25]	1036	1.07
gi|229364725|emb|CAY52696.1|	putative ABC transport system, outer membrane exported protein [Pseudomonas fluorescens SBW25]	1158	1.05
gi|229361206|emb|CAY48070.1|	succinyl-CoA synthetase beta chain [Pseudomonas fluorescens SBW25]	1119	1
gi|229364744|emb|CAY52723.1|	DNA-directed RNA polymerase alpha chain [Pseudomonas fluorescens SBW25]	1117	0.98
gi|229365349|emb|CAY53727.1|	ATP synthase alpha chain [Pseudomonas fluorescens SBW25]	813	0.92
gi|229364775|emb|CAY52776.1|	DNA-directed RNA polymerase beta~ chain [Pseudomonas fluorescens SBW25]	3251	0.89
gi|229360807|emb|CAY47665.1|	probable alcohol dehydrogenase [Pseudomonas fluorescens SBW25]	414	0.87
gi|229359883|emb|CAY46736.1|	pyruvate dehydrogenase E1 component [Pseudomonas fluorescens SBW25]	1453	0.86
gi|229360345|emb|CAY47202.1|	putative lipoprotein [Pseudomonas fluorescens SBW25]	1528	0.84
gi|229361207|emb|CAY48071.1|	succinyl-CoA synthetase alpha chain [Pseudomonas fluorescens SBW25]	199	0.81
gi|229364173|emb|CAY51832.1|	putative ferritin DPS-family DNA binding protein [Pseudomonas fluorescens SBW25]	471	0.81
gi|229364764|emb|CAY52758.1|	50S ribosomal protein L22 [Pseudomonas fluorescens SBW25]	666	0.78
gi|229360239|emb|CAY47096.1|	putative exported protein [Pseudomonas fluorescens SBW25]	171	0.74
gi|229364385|emb|CAY52165.1|	putative aminotransferase [Pseudomonas fluorescens SBW25]	353	0.73
gi|229364776|emb|CAY52777.1|	DNA-directed RNA polymerase beta chain [Pseudomonas fluorescens SBW25]	1084	0.7
gi|229364115|emb|CAY51736.1|	putative exported protein [Pseudomonas fluorescens SBW25]	1362	0.7
gi|229363442|emb|CAY50639.1|	50S ribosomal protein L20 [Pseudomonas fluorescens SBW25]	79	0.68
gi|229364757|emb|CAY52745.1|	50S ribosomal protein L5 [Pseudomonas fluorescens SBW25]	446	0.67
gi|229360318|emb|CAY47175.1|	putative lipoprotein [Pseudomonas fluorescens SBW25]	308	0.67
gi|229365347|emb|CAY53725.1|	ATP synthase beta chain [Pseudomonas fluorescens SBW25]	258	0.66
gi|229360143|emb|CAY47000.1|	putative ribosomal protein L25p family [Pseudomonas fluorescens SBW25]	80	0.63
gi|229362326|emb|CAY49228.1|	putative aldo/keto reductase [Pseudomonas fluorescens SBW25]	281	0.62
gi|229360557|emb|CAY47414.1|	putative alkyl hydroperoxide reductase [Pseudomonas fluorescens SBW25]	130	0.62
gi|229364743|emb|CAY52721.1|	50S ribosomal protein L17 [Pseudomonas fluorescens SBW25]	83	0.62
gi|229363458|emb|CAY50667.1|	putative L-asparaginase II precursor [Pseudomonas fluorescens SBW25]	273	0.59
gi|229359800|emb|CAY46650.1|	glucans biosynthesis protein G precursor [Pseudomonas fluorescens SBW25]	310	0.58
gi|229360548|emb|CAY47405.1|	glycerol kinase [Pseudomonas fluorescens SBW25]	1309	0.58

gi|229359726|emb|CAY46574.1|	outer membrane protein, oprE3 precursor [Pseudomonas fluorescens SBW25]	795	0.58
gi|229359882|emb|CAY46735.1|	dihydrolipoyllysine-residue acetyltransferase component of pyruvate dehydrogenase complex [Pseudomonas fluorescens SB	962	0.57
gi|111111137|gb|EWC11137.1|	47132620_keratin_2_Homo_sapiens [contaminants]	1225	0.56
gi|229362018|emb|CAY48919.1|	putative ferric alcaligin siderophore receptor [Pseudomonas fluorescens SBW25]	437	0.55
gi|229361204|emb|CAY48068.1|	dihydrolipoyllysine-residue succinyltransferase component of 2-oxoglutarate dehydrogenase complex [Pseudomonas fluore	666	0.53
gi|229364712|emb|CAY52676.1|	6,7-dimethyl-8-ribityllumazine synthase [Pseudomonas fluorescens SBW25]	405	0.53
gi|229361847|emb|CAY48745.1|	putative TonB-receptor protein [Pseudomonas fluorescens SBW25]	1014	0.52
gi|229363503|emb|CAY50737.1|	aromatic-amino-acid aminotransferase [Pseudomonas fluorescens SBW25]	193	0.52
gi|229363585|emb|CAY50877.1|	putative putative heat shock protein A [Pseudomonas fluorescens SBW25]	640	0.52
gi|229363881|emb|CAY51363.1|	putative outer membrane porin A protein [Pseudomonas fluorescens SBW25]	910	0.51
gi|229360663|emb|CAY47521.1|	putative lysine biosynthesis-related protein [Pseudomonas fluorescens SBW25]	216	0.49
gi|229364778|emb|CAY52781.1|	50S ribosomal protein L10 [Pseudomonas fluorescens SBW25]	180	0.49
gi|229360407|emb|CAY47264.1|	putative amino acid ABC transport system, substrate-binding protein [Pseudomonas fluorescens SBW25]	204	0.48
gi|229363134|emb|CAY50159.1|	conserved hypothetical protein [Pseudomonas fluorescens SBW25]	79	0.48
gi|229359915|emb|CAY46769.1|	putative lipoprotein [Pseudomonas fluorescens SBW25]	104	0.47
gi|229361044|emb|CAY47906.1|	polysaccharide biosynthesis protein [Pseudomonas fluorescens SBW25]	250	0.45
gi|229360636|emb|CAY47494.1|	putative lipoprotein [Pseudomonas fluorescens SBW25]	278	0.45
gi|229360153|emb|CAY47010.1|	putative exported protein [Pseudomonas fluorescens SBW25]	68	0.45
gi|229363110|emb|CAY50120.1|	isocitrate dehydrogenase [NADP] [Pseudomonas fluorescens SBW25]	792	0.44
gi|229363660|emb|CAY51010.1|	diaminobutyrate--2-oxoglutarate aminotransferase [Pseudomonas fluorescens SBW25]	505	0.43
gi|229360235|emb|CAY47092.1|	putative outer membrane porin [Pseudomonas fluorescens SBW25]	418	0.43
gi|229365217|emb|CAY53511.1|	putative phosphomannomutase/phosphoglucomutase [Pseudomonas fluorescens SBW25]	694	0.43
gi|229364304|emb|CAY52041.1|	conserved hypothetical protein [Pseudomonas fluorescens SBW25]	218	0.42
gi|229360670|emb|CAY47528.1|	elongation factor [Pseudomonas fluorescens SBW25]	71	0.42
gi|229360462|emb|CAY47319.1|	cytosol aminopeptidase [Pseudomonas fluorescens SBW25]	661	0.41
gi|229360341|emb|CAY47198.1|	putative phospholipid-binding lipoprotein [Pseudomonas fluorescens SBW25]	183	0.41
gi|229362316|emb|CAY49218.1|	alkyl hydroperoxide reductase protein [Pseudomonas fluorescens SBW25]	93	0.41
gi|229364498|emb|CAY52345.1|	polynucleotide phosphorylase/polyadenylase [Pseudomonas fluorescens SBW25]	512	0.4
gi|229365218|emb|CAY53512.1|	acetylglutamate kinase [Pseudomonas fluorescens SBW25]	130	0.4
gi|229362266|emb|CAY49168.1|	putative TonB-dependent receptor YncD precursor [Pseudomonas fluorescens SBW25]	813	0.39
gi|229359623|emb|CAY46465.1|	4-aminobutyrate aminotransferase [Pseudomonas fluorescens SBW25]	162	0.38
gi|229364518|emb|CAY52373.1|	chaperone protein DnkA (HSP70) [Pseudomonas fluorescens SBW25]	396	0.37
gi|229363109|emb|CAY50118.1|	isocitrate dehydrogenase [NADP] [Pseudomonas fluorescens SBW25]	584	0.37
gi|229360144|emb|CAY47001.1|	ribose-phosphate pyrophosphokinase [Pseudomonas fluorescens SBW25]	117	0.37
gi|229364178|emb|CAY51840.1|	putative family S43 non-peptidase homologue [Pseudomonas fluorescens SBW25]	645	0.36
gi|229359946|emb|CAY46800.1|	adenylosuccinate synthetase [Pseudomonas fluorescens SBW25]	157	0.36
gi|229360640|emb|CAY47498.1|	adenylate kinase [Pseudomonas fluorescens SBW25]	56	0.36
gi|229360146|emb|CAY47003.1|	putative outer-membrane lipoprotein [Pseudomonas fluorescens SBW25]	318	0.36
gi|229359527|emb|CAY46368.1|	thiol:disulfide interchange protein [Pseudomonas fluorescens SBW25]	59	0.36
gi|229360305|emb|CAY47162.1|	ATP phosphoribosyltransferase [Pseudomonas fluorescens SBW25]	47	0.36

gi|229364468|emb|CAY52297.1|	ketol-acid reductoisomerase [Pseudomonas fluorescens SBW25]	252	0.35
gi|229364492|emb|CAY52336.1|	glucose-6-phosphate isomerase [Pseudomonas fluorescens SBW25]	252	0.34
gi|229364100|emb|CAY51711.1|	putative porin [Pseudomonas fluorescens SBW25]	497	0.34
gi|229364995|emb|CAY53133.1|	aspartate carbamoyltransferase [Pseudomonas fluorescens SBW25]	116	0.34
gi|229359606|emb|CAY46448.1|	putative lipoprotein [Pseudomonas fluorescens SBW25]	39	0.34
gi|229362693|emb|CAY49603.1|	putative ferric alcaligin siderophore receptor [Pseudomonas fluorescens SBW25]	388	0.32
gi|229359775|emb|CAY46625.1|	glutamine synthetase [Pseudomonas fluorescens SBW25]	557	0.32
gi|229365298|emb|CAY53646.1|	oxaloacetate decarboxylase alpha chain [Pseudomonas fluorescens SBW25]	199	0.32
gi|229359622|emb|CAY46464.1|	succinate-semialdehyde dehydrogenase [NADP+] [Pseudomonas fluorescens SBW25]	246	0.32
gi|229363488|emb|CAY50712.1|	putative 3-isopropylmalate dehydrogenase [Pseudomonas fluorescens SBW25]	171	0.32
gi|229360669|emb|CAY47527.1|	30S ribosomal protein S2 [Pseudomonas fluorescens SBW25]	42	0.3
gi|229359476|emb|CAY46317.1|	galactonate dehydratase [Pseudomonas fluorescens SBW25]	203	0.29
gi|229364771|emb|CAY52770.1|	elongation factor TU [Pseudomonas fluorescens SBW25]	845	0.28
gi|229364029|emb|CAY51593.1|	putative histidine-binding periplasmic protein precursor [Pseudomonas fluorescens SBW25]	55	0.28
gi|229364672|emb|CAY52606.1|	gamma-glutamyl phosphate reductase [Pseudomonas fluorescens SBW25]	313	0.27
gi|229360689|emb|CAY47547.1|	enolase [Pseudomonas fluorescens SBW25]	202	0.27
gi|229360561|emb|CAY47418.1|	argininosuccinate synthase [Pseudomonas fluorescens SBW25]	120	0.27
gi|229364772|emb|CAY52772.1|	elongation factor G [Pseudomonas fluorescens SBW25]	225	0.26
gi|229363756|emb|CAY51170.1|	putative fatty acid transport system, membrane protein [Pseudomonas fluorescens SBW25]	496	0.26
gi|229362428|emb|CAY49334.1|	putative carboxyvinyl-carboxyphosphonate phosphorylmutase [Pseudomonas fluorescens SBW25]	54	0.26
gi|229364659|emb|CAY52587.1|	putative lipoprotein [Pseudomonas fluorescens SBW25]	276	0.25
gi|229360284|emb|CAY47141.1|	fumarate hydratase class II [Pseudomonas fluorescens SBW25]	254	0.25
gi|229364606|emb|CAY52504.1|	putative TonB-dependent receptor protein [Pseudomonas fluorescens SBW25]	408	0.24
gi|229360973|emb|CAY47833.1|	transaldolase B [Pseudomonas fluorescens SBW25]	70	0.24
gi|229365299|emb|CAY53647.1|	biotin carboxylase [Pseudomonas fluorescens SBW25]	136	0.23
gi|229359912|emb|CAY46766.1|	putative outer membrane protein [Pseudomonas fluorescens SBW25]	74	0.23
gi|229362643|emb|CAY49551.1|	putative rhizopine biosynthesis/dihydropicolinate synthase [Pseudomonas fluorescens SBW25]	109	0.23
gi|229361213|emb|CAY48077.1|	chaperone (heat shock protein) [Pseudomonas fluorescens SBW25]	168	0.22
gi|229364218|emb|CAY51902.1|	glyceraldehyde 3-phosphate dehydrogenase 1 [Pseudomonas fluorescens SBW25]	65	0.22
gi|229363644|emb|CAY50984.1|	putative allantoicase [Pseudomonas fluorescens SBW25]	60	0.22
gi|229360942|emb|CAY47802.1|	fatty oxidation complex alpha subunit [Pseudomonas fluorescens SBW25]	246	0.21
gi|229363775|emb|CAY51194.1|	conserved hypothetical protein [Pseudomonas fluorescens SBW25]	127	0.21
gi|229361927|emb|CAY48827.1|	putative TonB-dependent receptor [Pseudomonas fluorescens SBW25]	245	0.2
gi|229362479|emb|CAY49385.1|	phosphoglucomutase [Pseudomonas fluorescens SBW25]	99	0.2
gi|229360249|emb|CAY47106.1|	NADP(H)-dependt aldo-keto reductase [Pseudomonas fluorescens SBW25]	75	0.2
gi|229360742|emb|CAY47600.1|	branched amino acid ABC transport system, substrate-binding protein [Pseudomonas fluorescens SBW25]	53	0.2
gi|229361031|emb|CAY47893.1|	phosphoserine aminotransferase [Pseudomonas fluorescens SBW25]	43	0.2
gi|229362324|emb|CAY49226.1|	putative 4-hydroxyphenylpyruvate dioxygenase [Pseudomonas fluorescens SBW25]	39	0.2
gi|229360794|emb|CAY47652.1|	periplasmic beta-glucosidase precursor [Pseudomonas fluorescens SBW25]	93	0.19
gi|229360447|emb|CAY47304.1|	iron (III) dicitrate transport protein precursor [Pseudomonas fluorescens SBW25]	74	0.19

gi|229362518|emb|CAY49425.1|	3-oxoacyl-[acyl-carrier-protein] synthase II [Pseudomonas fluorescens SBW25]	370	0.19
gi|229360943|emb|CAY47803.1|	3-ketoacyl-CoA thiolase [Pseudomonas fluorescens SBW25]	266	0.19
gi|229362548|emb|CAY49455.1|	putative secreted pectate lyase [Pseudomonas fluorescens SBW25]	152	0.19
gi|229360870|emb|CAY47729.1|	putative lipoprotein [Pseudomonas fluorescens SBW25]	124	0.19
gi|229360677|emb|CAY47535.1|	putative surface exported protein [Pseudomonas fluorescens SBW25]	112	0.18
gi|229360332|emb|CAY47189.1|	conserved hypothetical protein [Pseudomonas fluorescens SBW25]	530	0.18
gi|229360121|emb|CAY46975.1|	putative lipoprotein [Pseudomonas fluorescens SBW25]	40	0.18
gi|229364996|emb|CAY53136.1|	dihydroorotase-like protein [Pseudomonas fluorescens SBW25]	167	0.18
gi|229363740|emb|CAY51145.1|	aspartate aminotransferase [Pseudomonas fluorescens SBW25]	149	0.18
gi|229361218|emb|CAY48082.1|	3-oxoacyl-[acyl-carrier-protein] synthase I [Pseudomonas fluorescens SBW25]	121	0.18
gi|229359832|emb|CAY46682.1|	putative NADP-dependent malic enzyme [Pseudomonas fluorescens SBW25]	248	0.17
gi|229364144|emb|CAY51781.1|	arginine deiminase [Pseudomonas fluorescens SBW25]	154	0.17
gi|229363227|emb|CAY50310.1|	ATP-dependent specificity component of clpP serine protease, chaperone [Pseudomonas fluorescens SBW25]	140	0.17
gi|229363655|emb|CAY51001.1|	putative aspartyl aminopeptidase [Pseudomonas fluorescens SBW25]	81	0.17
gi|229364576|emb|CAY52461.1|	serine hydroxymethyltransferase [Pseudomonas fluorescens SBW25]	55	0.17
gi|229360931|emb|CAY47791.1|	aconitate hydratase 1 [Pseudomonas fluorescens SBW25]	127	0.16
gi|229364030|emb|CAY51594.1|	acetyl-coenzyme A synthetase [Pseudomonas fluorescens SBW25]	87	0.16
gi|229361205|emb|CAY48069.1|	dihydrolipoyl dehydrogenase [Pseudomonas fluorescens SBW25]	146	0.16
gi|229363866|emb|CAY51338.1|	guanine deaminase [Pseudomonas fluorescens SBW25]	58	0.16
gi|229363123|emb|CAY50142.1|	NADH dehydrogenase I chain F [Pseudomonas fluorescens SBW25]	36	0.16
gi|229364150|emb|CAY51790.1|	glycine dehydrogenase [decarboxylating] [Pseudomonas fluorescens SBW25]	407	0.15
gi|229365287|emb|CAY53626.1|	aspartate ammonia-lyase [Pseudomonas fluorescens SBW25]	137	0.15
gi|229364959|emb|CAY53075.1|	adenosylhomocysteinase [Pseudomonas fluorescens SBW25]	80	0.15
gi|229365209|emb|CAY53495.1|	putative aldehyde dehydrogenase [Pseudomonas fluorescens SBW25]	51	0.15
gi|229362242|emb|CAY49144.1|	CDP-diacylglycerol-serine O-phosphatidyltransferase [Pseudomonas fluorescens SBW25]	46	0.15
gi|229360333|emb|CAY47190.1|	putative exported protein [Pseudomonas fluorescens SBW25]	43	0.15
gi|229362536|emb|CAY49443.1|	putative diaminobutyrate--2-oxoglutarate aminotransferase [Pseudomonas fluorescens SBW25]	38	0.15
gi|229360401|emb|CAY47258.1|	conserved hypothetical protein [Pseudomonas fluorescens SBW25]	35	0.15
gi|229362669|emb|CAY49579.1|	putative amidase [Pseudomonas fluorescens SBW25]	162	0.14
gi|229364641|emb|CAY52559.1|	AMP nucleosidase [Pseudomonas fluorescens SBW25]	418	0.14
gi|229363888|emb|CAY51376.1|	putative phosphoenolpyruvate synthase [Pseudomonas fluorescens SBW25]	203	0.13
gi|229364293|emb|CAY52028.1|	GMP synthase (glutamine-hydrolyzing) [Pseudomonas fluorescens SBW25]	55	0.13
gi|229359950|emb|CAY46804.1|	ribonuclease R [Pseudomonas fluorescens SBW25]	82	0.12
gi|229360147|emb|CAY47004.1|	conserved hypothetical protein [Pseudomonas fluorescens SBW25]	97	0.12
gi|229365348|emb|CAY53726.1|	ATP synthase gamma chain [Pseudomonas fluorescens SBW25]	67	0.12
gi|229361036|emb|CAY47898.1|	30S ribosomal protein S1 [Pseudomonas fluorescens SBW25]	58	0.12
gi|229365033|emb|CAY53201.1|	dihydroxy-acid dehydratase [Pseudomonas fluorescens SBW25]	45	0.12
gi|229361242|emb|CAY48106.1|	putative dipeptidase [Pseudomonas fluorescens SBW25]	39	0.12
gi|229364170|emb|CAY51826.1|	aspartyl-tRNA synthetase [Pseudomonas fluorescens SBW25]	36	0.12
gi|229361203|emb|CAY48067.1|	2-oxoglutarate dehydrogenase E1 component [Pseudomonas fluorescens SBW25]	128	0.11

gi|229360459|emb|CAY47316.1|	valyl-tRNA synthetase [Pseudomonas fluorescens SBW25]	62	0.11
gi|229361399|emb|CAY48274.1|	tail-specific protease precursor [Pseudomonas fluorescens SBW25]	59	0.1
gi|229359454|emb|CAY46295.1|	glycyl-tRNA synthetase beta chain (ec 6.1.1.14) (glycine--trna ligase beta chain) (glyrs) [Pseudomonas fluorescens SBW25]	59	0.1
gi|229362684|emb|CAY49594.1|	putative glycogen debranching protein [Pseudomonas fluorescens SBW25]	65	0.09
gi|229364285|emb|CAY52013.1|	phosphoribosylformylglycinamidine synthase [Pseudomonas fluorescens SBW25]	304	0.08
gi|229360194|emb|CAY47051.1|	chaperone [Pseudomonas fluorescens SBW25]	66	0.08
gi|229360639|emb|CAY47497.1|	phosphoenolpyruvate carboxylase [Pseudomonas fluorescens SBW25]	55	0.08
gi|229362012|emb|CAY48913.1|	putative aminopeptidase N [Pseudomonas fluorescens SBW25]	31	0.08
gi|229364817|emb|CAY52842.1|	putative exported organic solvent tolerance protein [Pseudomonas fluorescens SBW25]	141	0.07
